# Fully Transparent, Ultrathin Flexible Organic Electrochemical Transistors with Additive Integration for Bioelectronic Applications

**DOI:** 10.1002/advs.202204746

**Published:** 2022-11-14

**Authors:** Ashuya Takemoto, Teppei Araki, Kazuya Nishimura, Mihoko Akiyama, Takafumi Uemura, Kazuki Kiriyama, Johan M. Koot, Yuko Kasai, Naoko Kurihira, Shuto Osaki, Shin‐ichi Wakida, Jaap M.J. den Toonder, Tsuyoshi Sekitani

**Affiliations:** ^1^ The Institute of Scientific and Industrial Research (SANKEN) Osaka University Ibaraki 567‐0047 Japan; ^2^ Department of Applied Physics Graduate School of Engineering Osaka University Suita 565‐0871 Japan; ^3^ Advanced Photonics and Biosensing Open Innovation Laboratory AIST‐Osaka University Suita 565‐0871 Japan; ^4^ Department of Mechanical Engineering and Institute for Complex Molecular Systems Eindhoven University of Technology Eindhoven 5600 MB The Netherlands

**Keywords:** bioelectronic sensors, flexible electronics, transparent devices

## Abstract

Optical transparency is highly desirable in bioelectronic sensors because it enables multimodal optical assessment during electronic sensing. Ultrathin (<5 µm) organic electrochemical transistors (OECTs) can be potentially used as a highly efficient bioelectronic transducer because they demonstrate high transconductance during low‐voltage operation and close conformability to biological tissues. However, the fabrication of fully transparent ultrathin OECTs remains a challenge owing to the harsh etching processes of nanomaterials. In this study, fully transparent, ultrathin, and flexible OECTs are developed using additive integration processes of selective‐wetting deposition and thermally bonded lamination. These processes are compatible with Ag nanowire electrodes and conducting polymer channels and realize unprecedented flexible OECTs with high visible transmittance (>90%) and high transconductance (≈1 mS) in low‐voltage operations (<0.6 V). Further, electroencephalogram acquisition and nitrate ion sensing are demonstrated in addition to the compatibility of simultaneous assessments of optical blood flowmetry when the transparent OECTs are worn, owing to the transparency. These feasibility demonstrations show promise in contributing to human stress monitoring in bioelectronics.

## Graphical abstract

An organic electrochemical transistor that enables multifaceted measurement of electric potential, light, and ions with low voltage operation has been developed toward human stress assessment. The transistor is precisely fabricated by an additive process that reduces damage to nanomaterials, resulting in ultrathin, flexible, and transparent sensor sheets. Contribution to saving resources, power, and footprint would be expected.

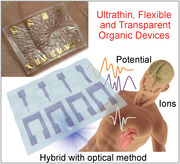

## 1 Introduction

Optically transparent and mechanically flexible sensors are of paramount importance in bioelectronics,^[^ ^1^ ^]^ such as electronic skins,^[^ ^2^, ^3^, ^4^ ^]^ neural interfaces,^[^ ^5^, ^6^, ^7^, ^8^ ^]^ user‐interactive displays,^[^ ^9^, ^10^, ^11^, ^12^ ^]^ and smart contact lenses.^[^ ^13^, ^14^ ^]^ In contrast to conventional opaque flexible electronics, their high visible transmittance permits the optical recording and manipulation of biological activities during electrical assessment^[^ ^5^, ^6^, ^7^, ^8^, ^15^ ^]^ and shows the sensed data through displays or color‐changing materials.^[^ ^9^, ^10^, ^11^, ^12^, ^14^, ^16^ ^]^ Multimodal functionalities are essential in bioelectronics for complex point‐of‐care diagnoses. As a specific case, human stress monitoring requires the identification of various vital signals, such as electroencephalogram, blood flow, and body fluid analysis, simultaneously and/or at the same location for accurate diagnosis.^[^ ^17^, ^18^ ^]^ As these signals are obtained using a combination of electrical, optical, and chemical methods, transparent flexible sensors are suitable for human stress monitoring owing to their multimodal accessibility.

For the construction of transparent flexible sensors, solution‐processable nanomaterials, such as metal nanowires,^[^ ^19^ ^]^ carbon nanotubes (CNTs),^[^ ^20^ ^]^ and graphene,^[^ ^21^ ^]^ have garnered significant attention owing to their mechanical flexibility, which helps in reducing the mechanical mismatch with biological tissues and high optoelectrical performance (sheet resistance <30 Ω sq^−1^, visible transmittance >90%), which is comparable to that of conventional transparent electrodes (i.e., brittle metal oxides). For example, transparent electrodes based on these nanomaterials can be directly attached to biological tissues and acquire electrophysiological signals simultaneously with optical observations and/or manipulation, resulting in a more accurate evaluation of complex biological activities.^[^ ^5^, ^6^, ^7^, ^8^ ^]^ Furthermore, the solution processability of these nanomaterials enables large‐area and low‐cost printing production for practical use in consumer electronics. Owing to these advantages, nanomaterial‐based transparent electrodes are becoming powerful tools for improving the optoelectronic sensor performance.

Although passive sensors with transparent electrodes are promising, transparent active components (i.e., transparent flexible transistors) should also be integrated within the system to ensure signal amplification and circuit applicability. To create such transistors, transparent active channels are required in addition to transparent flexible electrodes. Organic electrochemical transistors (OECTs), whose active channels include transparent organic mixed ionic/electronic conductors, such as poly(3,4‐ethylenedioxythiophene) doped with poly(styrene sulfonate) (PEDOT:PSS), are a promising candidate because they can achieve high transconductance at an order of millisiemens, even during low‐voltage operation (<0.6 V); thus, presenting their favorable properties for bioelectronic systems.^[^ ^22^ ^]^ Remarkably, the reported transconductance is higher than that of other transparent channel materials such as semiconducting metal oxides (ZnO, n‐InAs, and GaN/InAlN), CNTs, and graphene.^[^ ^23^ ^]^ Recently, flexible OECTs were developed on ultrathin (<5 µm thick) polymer films to optimize the fabrication process,^[^ ^23^, ^24^, ^25^, ^26^, ^27^ ^]^ leading to conformable neural interfaces^[^ ^25^, ^26^, ^28^ ^]^ and self‐powered flexible biosensors.^[^ ^27^, ^29^ ^]^ The favorable properties and developed fabrication processes offer prominent opportunities for creating transparent flexible OECTs; however, they are incompatible with solution‐processable nanomaterials for transparent flexible source/drain electrodes. This is because the etching procedures that use reactive ions and photolithography can oxidize or decompose nanomaterials such as metal nanowires and nanocarbons. Therefore, the source/drain electrodes in ultrathin OECTs are limited to Au (inactive metal), which exhibits lower transmittance.

**Figure 1:**
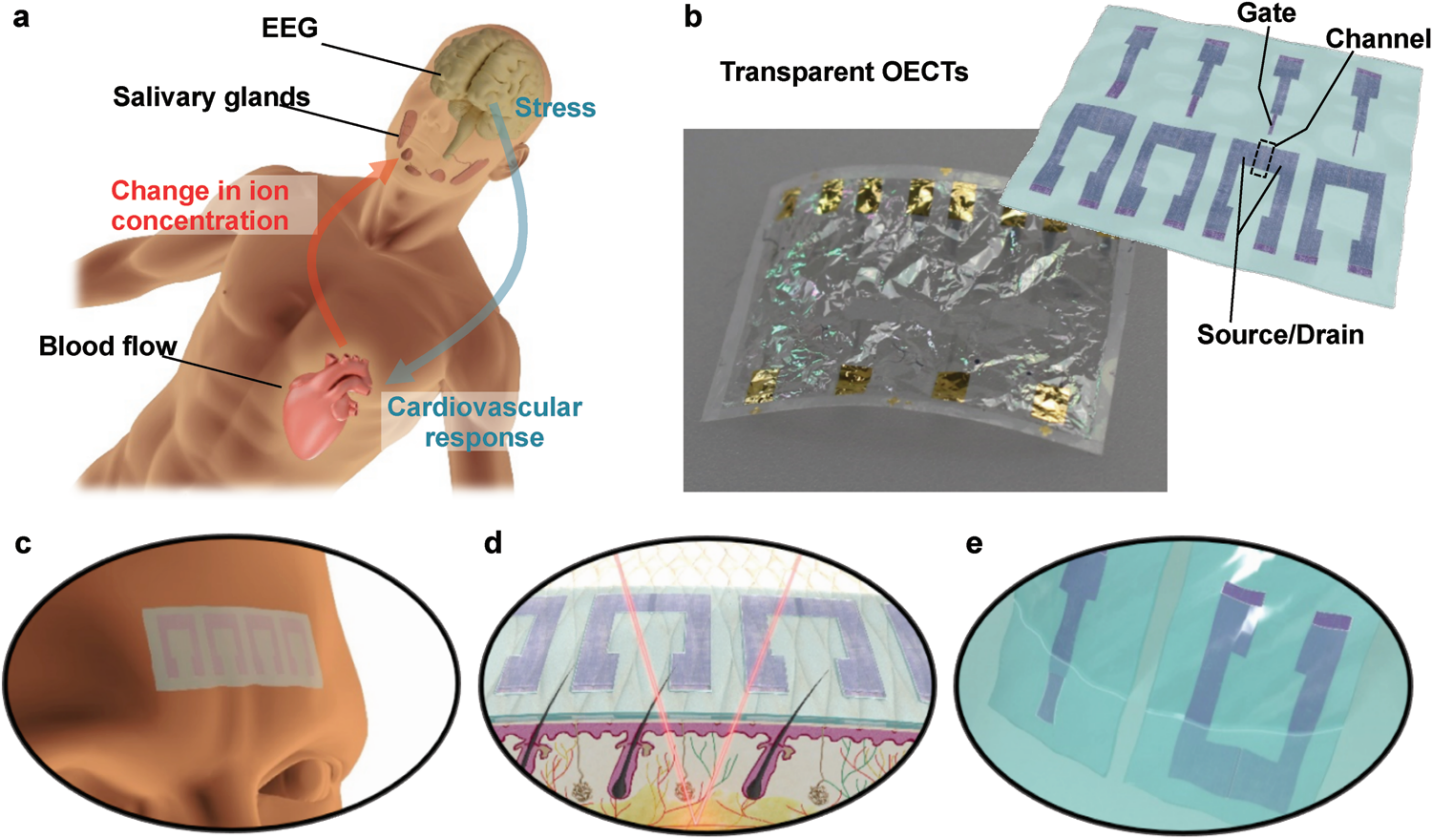
Stress monitoring adaptation of OECTs. a) The flow from stress response to nitrate secretion; when the brain, the center of the autonomic nervous system, responds to stress, blood flow decreases due to vasoconstrictive effects, followed by nitric oxide production and metabolite nitrate ion secretion for vascular relaxation. b–e) Fully‐transparent ultrathin OECTs (b) show measurement compatibility for EEG (c), blood flow (d), and nitrate ion concentration (e), which are necessary for stress monitoring. In (b), the contact pads that are connected to the gate/source/drain electrodes of fully‐transparent ultrathin OECTs are located near the edge of the substrate and are fabricated using evaporated Au (thickness: 50 nm).

In this study, we developed fully transparent flexible OECTs for future application in human stress monitoring and proposed additive fabrication processes to integrate highly transparent silver nanowire (AgNW) electrodes and conducting polymer channels (**Figure** **1**; Figure S1, Supporting Informatiob). In contrast to previous etching‐based processes, our processes include a selective wetting deposition technique and thermal lamination method, enabling deposition and patterning simultaneously to ensure high‐performance maintenance of constituent materials (AgNW electrodes, PEDOT:PSS channels, and 1 µm thick encapsulation layers with hole patterns). As these processes do not use harsh chemicals or reactive ions, the electrical properties of AgNW source/drain electrodes with high visible transmittance (>90%) can be preserved. Such high transparency is unprecedented for OECTs so far; however, high transconductance was obtained at an order of millisiemens (≈1 mS), comparable to that of typical OECTs using Au source/drain electrodes. The electrical performance shows the effectiveness of the proposed processes in achieving state‐of‐the‐art performance for OECTs. Furthermore, they exhibit high conformability to the skin owing to the ultra‐thinness (2 µm thick) of OECTs, which is favorable for wearable applications. To demonstrate the sensor application, ultrathin transparent OECTs were used for electroencephalography (EEG) and nitrate ion sensing; additionally, simultaneous optical sensing was included for blood flow directly above the OECTs (Figure 1c–e). These sensing targets are correlated with psychological states for mental stress monitoring or human–machine interfaces. These demonstrations suggest a novel possibility for multimodal assessment using electrophysiological, optical, and ionic methods, which have not yet been integrated into a single device.

## 2 Structure of Fully Transparent Ultrathin OECTs

**Figure 2:**
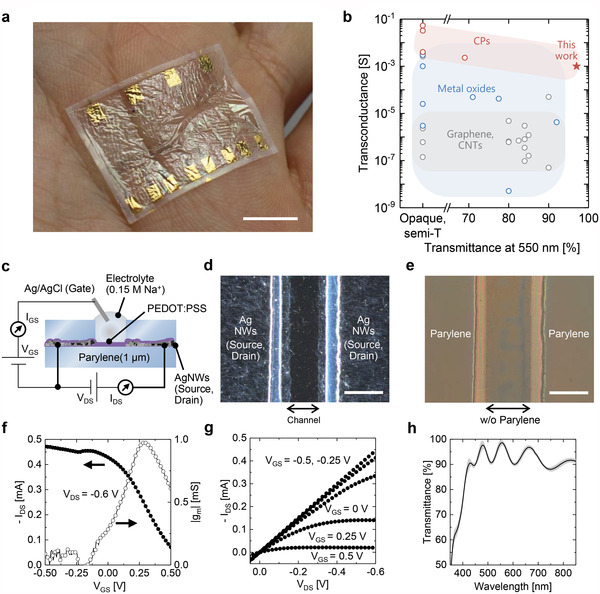
Fully transparent ultrathin OECTs. a) Photograph of fully transparent ultrathin OECTs conformable to the skin. The scale bar corresponds to 1 cm. As shown in Figure 1b, the contact pads are fabricated using evaporated Au along the edge of the substrate to measure the fully transparent ultrathin OECT. b) Transconductance as a function of optical transmittance (at a wavelength of 550 nm) of the developed OECTs and transparent transistors (see the Experimental Section for details). The transmittance corresponds to the source/drain electrodes, excluding substrate and encapsulation. If not apparent, the remarked values are plotted. c–e) Cross‐sectional illustrations of OECTs via wiring diagram (c) and optical micrographs under dark (d) and bright fields (e). Scale bars correspond to 100 µm. f,g) Transfer (f) and output (g) characteristics of representative OECTs with channel length and width of 100 and 2000 µm, respectively. h) Optical transmittance spectrum of AgNWs‐PEDOT:PSS in the visible wavelength. The shaded region represents a standard deviation in six samples.

**Figure** **2** illustrates the fully transparent 2 µm thick OECT device. Such devices with a thickness of <5 µm have the advantage of high conformability to biological tissues.^[^ ^25^, ^27^, ^30^ ^]^ Figure 2d,e shows the optical micrographs of OECTs under dark and bright fields, respectively, presenting the high process accuracy (i.e., the holes are clearly aligned on the channels). This aligned encapsulation layer ensures the operation of OECTs, as explained later. The fabrication processes of fully transparent ultrathin OECTs are based on additive integration, as shown in Figure S1, Supporting Information. The source/drain electrodes were patterned as cross‐aligned AgNW networks on 1 µm thick parylene substrates via selective wetting deposition, according to previous studies.^[^ ^31^, ^32^ ^]^ The patterning process used a rod coating suitable for large‐area manufacturing; nevertheless, it enabled the high‐resolution deposition/separation of high‐performance AgNW electrodes down to a width of 20 µm. Here, the AgNW electrodes were effectively separated with a spacing of 20 µm for channel definition, which was useful for scaling the OECTs. The patterned source/drain electrodes exhibited a high visible transmittance of 97% and low sheet resistance of 25 Ω per sq. owing to the high optoelectrical performance of AgNWs. The channels comprised PEDOT:PSS patterned using the same process as AgNW electrodes. In this channel formation, the AgNW electrodes (source/drain) were overcoated with PEDOT:PSS (≈180 nm thick), which could improve the conductivity^[^ ^33^ ^]^ with less effect on the optical transparency (Figure 2h). For the electrical insulation of the source/drain against electrolytes, the encapsulation layer with openings on the channels was bonded via the thermal lamination of 1 µm parylene films with hole patterns (Figures S2–S4, Supporting Information). Various narrow opening designs can be developed with a width of 20 µm. Moreover, the adhesion performance on the lamination exhibited a shear bonding strength of ≈0.3 MPa, which was sufficiently high for the ultrathin parylene substrate in terms of mechanical integrity (see Figures S5 and S6, Supporting Information). Saline solutions and Ag/AgCl electrodes were used as the ion source (i.e., electrolyte) and gate electrodes, respectively.

## 3 Electrical and Optical Characteristics Under Steady State

The representative electrical characteristics of fully transparent OECTs are shown in Figure 2f,g. The OECTs operated in the depletion mode, similar to conventional OECTs based on PEDOT:PSS;^[^ ^22^ ^]^ the drain current (*I*_DS_) decreased when the gate voltage was applied from −0.2 to 0.5 V. The champion data of the transfer characteristics (Figure 2f) exhibited high transconductance, ≈1 mS, which peaked at a gate voltage (*V*_GS_) of 0.25 V. The high transconductance was comparable to that of opaque OECTs prepared using Au electrodes.^[^ ^23^ ^]^ The OECTs also exhibited a visible transmittance of ≈90% in all regions of encapsulation and substrate, source/drain, and channel (see Figure S7, Supporting Information) because they were constructed using highly transparent source/drain electrodes with ≈97% transmittance owing to the proposed fabrication processes (Figure 2h; Figure S7, Supporting Information). To the best of our knowledge, these simultaneous functionalities have not yet been implemented in previous OECTs (Figure 2b; Section 10.4 explains the development of the figure and provides a detailed description). This efficient current modulation and high transparency can be attributed to the low sheet resistance of transparent AgNW electrodes (source/drain) and their effective insulation by the encapsulation layer. If the encapsulation layer is excluded or partially delaminated, the applied gate voltage activates electrochemical reactions between the source/drain and electrolyte; subsequently, current modulation in the channel becomes dysfunctional.

## 4 Scalability of OECTs and Electrical Performance

**Figure 3:**
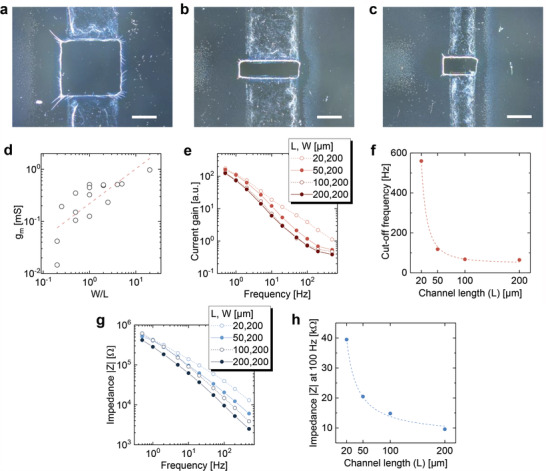
Scaling of fully transparent ultrathin OECTs. a–c) Dark‐field optical micrographs of OECTs with channel width/length (*W*/*L*) = 200/200 µm (a), 200/50 µm (b), and 100/50 µm (c). Scale bars correspond to 100 µm. d) Maximum transconductance as a function of channel geometries. The channel thickness is ≈180 nm. The red dashed line illustrates the linear regression for eye guides. e) Current gain ( = Δ*I*_DS_/Δ*I*_GS_) of OECTs with different *L* as a function of frequency. The measurements were conducted at *V*_DS_ = −0.6 V and *V*_GS_ = 10 mV amplitude sine wave. f) Cutoff frequency of OECTs with *W* = 200 µm as a function of *L*. The red dashed line corresponds to ≈1/*L* ^2^ fit (*R* ^2^ ≈ 0.99). g) Frequency plots of impedance (Δ*V*_GS_/Δ*I*_GS_) of OECTs (*W* = 200 µm) with different *L* = 20–200 µm. h) Impedance at 100 Hz as a function of *L*. The blue dashed line corresponds to ≈1/*L* fit (*R* ^2^ ≈ 0.99).

In the proposed fabrication processes, the size of OECTs can be reduced to a channel length (*L*) and width (*W*) of 20 µm, as shown in **Figure** **3**a–c; Figure S8, Supporting Information. To confirm the scaling effects on the electrical performance of OECTs, we first plotted the maximum transconductance of OECTs with different channel dimensions, as shown in Figure 3d; the transconductance increases with the ratio of *W*/*L*; however, it becomes saturated at larger *W*/*L* owing to the parasitic effects of contact or electrode resistance. We also characterized the frequency responses of OECTs with different channel lengths (20–200 µm). Figure 3e,f shows the frequency characterization of the current gain (Δ*I*_DS_/Δ*I*_GS_; *I*_GS_ is the gate current) of OECTs. The cutoff frequency of OECTs is extracted at Δ*I*_DS_/Δ*I*_GS_ = 1, which exhibits the maximum frequency to enable signal amplification (see Figure S9, Supporting Information). The current gain over the entire frequency range (0.5–500 Hz) increases as the channel length decreases; Figure 3f shows that the cutoff frequency increases from 64 to 560 Hz when the channel length decreases from 200 to 20 µm. These results indicate an improved frequency response with a smaller channel length, which is a reasonable trend for transistor scaling. It is noteworthy that the cutoff frequency varies according to 1/*L* ^2^, as stated in previous reports.^[^ ^34^, ^35^ ^]^ The impedance, *Z* ( = *v*_GS_/*i*_GS_), also depends on 1/*L* at the same channel width (i.e., area dependence of the capacitance), as shown in Figure 3g,h. These reasonably scaled parameters demonstrate the high‐dimensional accuracy of the proposed fabrication processes.

## 5 Evaluation of Non‐Responsiveness to Various Environments

**Figure 4:**
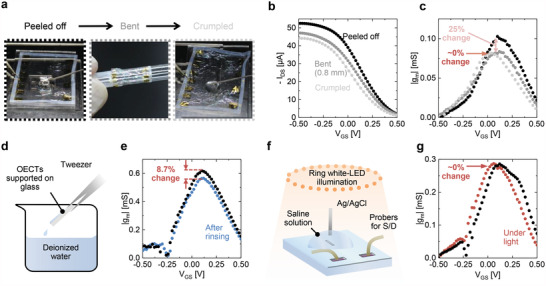
Characterization of OECTs under various environments. a–c) Microscopic photographs of OECTs (a), transfer characteristics (b), and the resulting transconductance (c) during mechanical deformation. The electrical characteristics are at *V*_DS_ = −0.6 V. d,e) Schematic of the rinsing procedure of OECTs (d) and the resulting transconductance before (black) and after (blue) rinsing (e). f,g) Schematic of OECT measurements under white‐LED illumination (f) and the resulting transconductance under dark environment (black) and white‐LED illumination (red) (g).

To evaluate the mechanical stability, we tested OECTs under mechanical deformations, as shown in **Figure** **4**a–c. In the first deformation into the bending state (diameter: 1.6 mm), the maximum transconductance decreased by 25%; however, the subsequent crumpling caused negligible changes (≈0%) in the transconductance (Figure 4c). The stability for subsequent deformation indicates a suitable function for sensing applications against dynamically moving biological tissues. The first decrease can be attributed to the relatively large deformation of PEDOT:PSS channels resulting from their position out of the neutral strain plane. This decrease can be reduced by reducing the channel areas for relatively small strain effects, filling the openings with solid‐state electrolytes, or engineering the device structures.

OECTs can be reused after rinsing the electrolytes with deionized water, as shown in Figure 4d,e; such rinsing capability is crucial for biosensing involving fluids. After rinsing for a minute, the maximum transconductance changed by 8% (Figure 4e), and the encapsulated parylene layer remained intact without delamination. These results indicate that the encapsulation layer effectively protects AgNW electrodes from delamination and short‐circuiting. Further, OECTs also leverage the capability of light‐insensitive operation under illumination from white light‐emitting diodes (LEDs), as depicted in Figure 4f,g. The gate voltage at peak transconductance was shifted by ≈0.01 V even under strong white light (irradiance of 2.3 mW cm^−2^), but the values around the peaks were similar (almost 0% change) at a certain gate voltage (≈0.06 V) in a dark environment and strong white light. Such light insensitivity is difficult in the low‐voltage operation of transparent organic field‐effect transistors, where transparent light‐insensitive organic semiconductors with a wide bandgap and high voltage operation must be selected.^[^ ^32^ ^]^ These results demonstrate that the developed transparent OECTs are useful for optoelectronic biological assessment with light manipulation and/or optical observations during electrical sensing.

## 6 EEG and Optical Assessments Using OECTs

**Figure 5:**
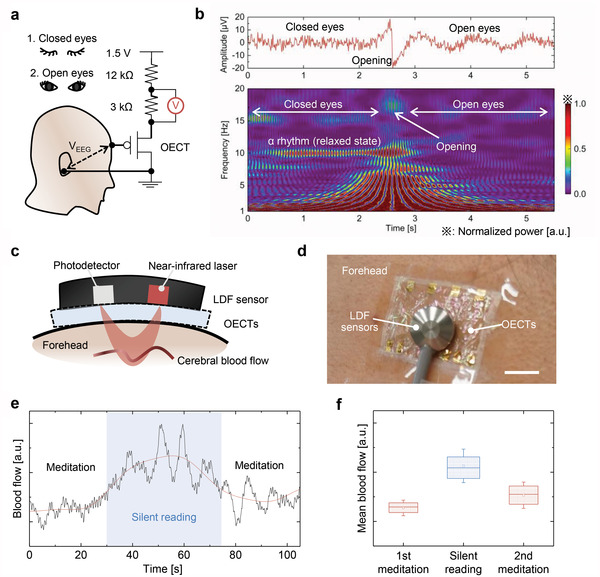
EEG acquisition and capability for simultaneous LDF. a) Schematic of measurement setup for EEG acquisition. The circuit diagram used when the PEDOT:PSS based channel is attached to the forehead, as shown in Figure 1c. Measurement was performed during closed and open eyes. b) Acquired traces in EEG during closed and open eyes (upper) and spectrogram analysis with Morlet wavelet (below). c) Cross‐sectional schematic and d) photograph of LDF sensing directly above the OECTs attached on the forehead, shown in Figure 1d. The scale bar corresponds to 1 cm. e) Acquired trace of blood flow (black line) in LDF sensing during meditation and silent reading. The red line indicates low‐pass‐filtered traces at <0.05 Hz for removing respiratory effects. f) Box plots of blood flow during meditation and silent reading in (e). The first and second meditation occurred during 0–30 s and 70–110 s, respectively. The blue‐shaded region in (e) indicates the silent reading period. The boxplots indicate the median (center line) and 25/75 percentiles (lowest and highest lines); square symbols and error bars indicate the mean value and standard deviation, respectively.

For the first demonstration of bioelectronic applications, EEG was performed with fully transparent ultrathin OECTs using a wireless module. EEG is a useful tool for the electrophysiological assessment of brain‐related conditions, such as mental stress, cognitive procedures, and neurological disorders, facilitating neurological applications, such as clinical diagnosis and brain–machine interfaces.^[^ ^36^ ^]^ Here, EEG signals were acquired from the human forehead before and after opening the eyes, as shown in **Figure** **5**a. In the acquisition setup, EEG signals were applied to the gate of OECTs, inducing drain‐current modulation. We used a serial resistor to translate the current modulation into voltage signals and detected it using the wireless module. Figure 5b shows the acquired traces and spectrograms of EEG signals before and after opening the eyes. While closing the eyes, alpha rhythms (8–10 Hz), which can be correlated with the relaxing state in humans,^[^ ^37^ ^]^ were clearly indicated; however, this indication weakened while opening the eyes owing to exiting the relaxation state (Figure 5b; Figure S10, Supporting Information). Alpha rhythm acquisition suggests that these OECTs can be used for electrophysiological assessment, such as mental health related to the relaxing state.

Furthermore, the multimodal assessment of more information about brain‐related conditions could be feasible with electrophysiological sensing using transparent OECTs. As shown in Figure 5c, laser Doppler flowmetry (LDF) was performed directly above the OECTs attached to the forehead for monitoring the blood flow; vertical placement directly above the electrophysiological sensors can improve the spatial resolution with a smaller footprint compared to the conventional in‐plane placement of EEG and LDF probes (Figure S11 and text, Supporting Information). Figure 5d shows the time traces of the recorded LDF signals before and after opening the eyes. As the OECTs effectively transmitted the near‐infrared laser (wavelength: 780 nm) owing to high transparency, LDF captured the decreased blood flow in the relaxing state, as previously reported.^[^ ^38^ ^]^ In addition, LDF data may be correlated with the aforementioned EEG data related to the relaxing state. These results suggest the capability of ultrathin transparent OECTs for simultaneous LDF with EEG, which can improve the accuracy of medical diagnosis^[^ ^39^, ^40^ ^]^ and classification of motor imagery.^[^ ^41^ ^]^

## 7 Nitrate Ion Sensing With OECTs

**Figure 6:**
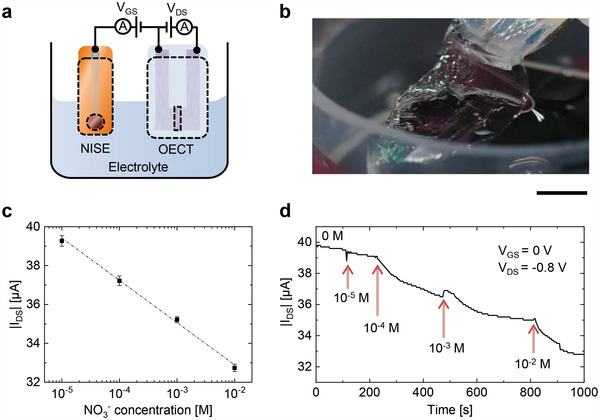
Nitrate ion sensing using OECTs. a) Schematic and b) photograph of the measurement setup for nitrate ions in electrolytes. Circuit diagram in (a) used during the immersion shown in Figure 1e. The scale bar corresponds to 0.5 cm. c) Measured steady‐state drain current (*I*_DS_) as a function of nitrate ion (NO_3_^−^) concentration in distilled water. d) Real‐time traces of *I*_DS_ in response to increased NO_3_^−^ concentration in distilled water at *V*_GS_ = 0 V and *V*_DS_ = −0.8 V.

OECTs demonstrated nitrate ion sensing capability, which can be a mental stress marker in biofluids.^[^ ^42^, ^43^ ^]^ When the brain, the center of the autonomic nervous system, responds to stress, blood flow decreases owing to vasoconstriction, followed by NO production and secretion of metabolite nitrate ions for vascular relaxation. In a previous report,^[^ ^43^ ^]^ increased nitrate levels in saliva suggested an acute stress state in humans, which may be correlated with stress‐induced gastric injury. Although EEG and LDF can suggest the stress/relaxing state in humans, nitrate ion sensing can offer further data for mental stress assessment, possibly related to stress‐induced organ diseases. **Figure** **6**a,b shows the measurement setup using OECTs for nitrate ion sensing. Nitrate ion selective electrodes were used as the gate electrodes to enhance sensitivity.^[^ ^44^ ^]^ As shown in Figure 6c,d, the source–drain current (*I*_DS_) steadily decreased with a sensitivity of 2.2 µA dec^−1^ when the nitrate concentration increased from 10^−5^ to 10^−2^ m in deionized water. Further, OECTs can detect the nitrate ion concentration (from 10^−5^ to 10^−2^ m) in electrolytes contaminated with other ion species (15 mm sodium chloride in this study) (Figure S12, Supporting Information). As the salivary nitrate level is 10^−5^ to10^−2^ m,^[^ ^45^ ^]^ OECTs suggest suitable functionality for potential stress monitoring.

## 8 Discussion

First, we discuss the reasons for the realization of fully transparent ultrathin OECTs with high transconductance. The key steps for this development include the additive layering fabrication processes, that is, selective wetting deposition and thermal lamination. Owing to the inertness of additive layering, these processes can avoid detrimental effects on functional materials (i.e., nanometals or nanocarbons) in OECT constructions, unlike conventional post‐etching‐based processes.^[^ ^23^, ^24^, ^25^, ^26^, ^27^ ^]^ Thus, ultrathin and transparent OECTs were successfully prepared with state‐of‐the‐art transparent electrodes (AgNW electrodes), which has not yet been achieved to the best of our knowledge (Figure 2b). The OECTs exhibited electrical characteristics comparable to those of conventional OECTs, along with unprecedentedly high optical transparency because the thermally laminated encapsulation layer effectively protected the high‐performance AgNW source/drain electrodes from corrosion, possibly caused by electrochemical reactions with the electrolytes.

Furthermore, during thermal lamination, the high shear bonding strength (≈0.3 MPa) ensured the mechanical integrity of devices for flexibility. The hole patterns on the encapsulation layer exhibited the same configuration before and after lamination (Figures S3 and S4, Supporting Information). This ideal lamination can be explained by the entanglement of polymer chains of parylene between the surfaces of encapsulation and substrate. A previous study^[^ ^46^ ^]^ on parylene bonding demonstrated that the bonding strength between thermally laminated parylene layers could be enhanced by increasing the heating temperature in the bonding process, which should be attributed to more activated movements in the polymer chains during heating and their subsequent entanglement under the bonding pressure. The parylene thermal expansion (≈3.5 × 10^−5^ °C^−1^)^[^ ^46^ ^]^ was small enough to conserve the configuration of hole patterns and thickness.

With the aim of realizing optoelectronic bio‐integrated systems, fully transparent ultrathin OECTs demonstrated sensing applications via EEG and simultaneous LDF. The acquired traces suggested a relaxing state in humans, presenting the possibility for the multimodal assessment of psychological signs^[^ ^39^ ^]^ (Figure 1). OECTs are also useful for ion sensing, presenting the opportunity to investigate biofluids (e.g., saliva) for additional information about psychological states.^[^ ^43^ ^]^ Such capability for multimodal assessment is favorable for enhancing diagnostic accuracy in neurological diseases^[^ ^39^, ^40^ ^]^ and improving the classification of motor imagery.^[^ ^41^ ^]^ Moreover, LDF probes can be placed directly above the OECTs owing to high transparency to improve the spatial resolution in the simultaneous sensing of electrophysiological and optical signals, which is crucial for understanding local biological activities (Figure S11, Supporting Information).

Furthermore, we verified the feasibility of simple circuits using the thin‐film transparent OECT developed in this study. The OECT was integrated into an inverter circuit that could be used for a switch circuit for light detection and luminescence, a voltage amplification to improve the signal‐to‐noise ratio, and a source follower circuit that can convert input/output impedance, all of which were successfully operated (Figures S13 and S14, Supporting Information). The transparent OECTs, which constituted these circuits, exhibited high bending durability down to a bending radius of 0.8 mm and under 1000 cyclic bending (Figure 4; and Figure S15, Supporting Information). The transparent flexible OECT is expected to be applied as a sensor element for stress‐free biological signal measurement on a daily basis in the future.

The current challenge in developing fully transparent and flexible electrochemical electronics is the connection points of the devices. Evaporated Au or printed Ag paste was deposited on contact pads of OECT, transparent AgNW electrodes, to evaluate OECT characteristics through external connections. However, the reinforced contact pads were opaque and inflexible (Figure 1b; Figure S15, Supporting Information). Transparent, stretchable, and printable adhesive electrode, recently reported, can become a candidate alternative material owing to a contact resistance of less than 100 Ω with transparent AgNW electrode having a sparse metal nanowire network.^[^ ^47^ ^]^ The contact resistance was sufficiently low compared with the electrode/skin contact impedance (1–100 kΩ) and did not affect the noise during electrophysiological recording. However, the high resistance at the connection point can be a major barrier in several applications, such as in improving the frequency characteristics for large transistor circuits. Further development of transparent and printable connection materials will be needed to achieve stable external connection terminals to realize fully transparent and flexible electronic circuits.

Although transparent flexible OECTs have been realized through conventional processes for bioelectronic sensors using microgrid‐patterned Au transparent electrodes in a previous study,^[^ ^26^ ^]^ the transparency and spatial resolution of transparent electrodes exhibit a trade‐off relationship defined by the grid size and Au‐line width (few tens of micrometers), resulting in larger (>100 µm width) total areas for the source/drain electrodes. In contrast, the proposed processes integrated AgNW transparent electrodes, which can be patterned down to a width of 20 µm or less via printing technologies.^[^ ^32^ ^]^ Furthermore, these processes can be applied to other solution‐processable nanomaterials for transparent electrodes, such as CNTs and graphene. The versatile options of the constituent materials can potentially improve the performance through downscaling or unprecedented material combinations in transparent flexible OECTs.

In this study, the OECT characteristics were evaluated using Ag/AgCl (probe type) as the gate electrode, as shown in Figures 2c and 4f, which is not suitable for the device downscaling. To enable the miniaturization of a fully transparent and flexible OECT, the gate should be in a planar structure, as shown in Figure 1b. Figure S16, Supporting Information, shows that a high transconductance of millisiemens (≈1 mS) was obtained with the planar structure; the results were equivalent to those with the Ag/AgCl gate electrode, as shown in Figure 2f. In the near future, the planar structure, which is compatible with the printing formation process, is expected to enable the integration of OECTs for fully transparent and flexible electronics.

## 9 Conclusion

In summary, we demonstrated transparent flexible active components for improving optoelectronic bio‐integrated systems by constructing ultrathin and transparent OECTs via the integration of additive layers. High transparency and favorable electrical characteristics were achieved because the proposed processes without harsh chemicals or post etching enabled the use of high‐performance transparent electrodes composed of AgNWs. These features facilitated electrophysiological and ion sensing along with the capability for simultaneous optical sensing. These results suggest that OECTs can detect psychological signs via electrophysiological, optical, and ionic measures, which can be applied to multimodal assessments for enhanced accuracy in understanding or classifying mental stress, biological activity, and motor imagery. These results will pave the way for more elaborate optoelectronic bio‐integrated systems.

## 10 Experimental Section

### Preparation of Dispersive Solutions and Electronic Materials

AgNW dispersion was acquired (Showa Denko K.K., JPN) and mixed with 50 wt% isopropanol for fine patterning the source and drain electrodes, as previously reported.^[^ ^31^, ^32^ ^]^ PEDOT:PSS dispersion was purchased (Orgacon N‐1005; Sigma–Aldrich Co. LLC., USA) and mixed with 0.5 wt% 3‐glycidoxypropyltrimethoxysilane (GOPS), 10 wt% ethylene glycol (EG), and 50 wt% isopropanol for fine patterning the active channels. Saline solution (Otsuka Pharmaceutical Co. Ltd., JPN) and Ag/AgCl electrodes (World Precision Instruments, USA) were purchased and used as the electrolyte and gate electrodes, respectively.

### Thermal Lamination of Parylene Films

Parylene films (diX‐SR; Daisankasei Co. Ltd., JPN) with a thickness of 1 µm were deposited on supporting glasses (Eagle‐XG; Corning Incorporated, USA) via chemical vapor deposition (CVD). To facilitate peeling off parylene films, the supporting glasses were pre‐coated with a fluorine carbon layer (Cytop; AGC Inc., JPN). Subsequently, a pair of parylene films was placed in contact with each other and pressed with a pressure of 5 MPa at 150 °C using a compact heating press (IMC‐180C; IMOTO MACHINERY Co. Ltd., JPN). After pressing for 20 min, the samples were released and cooled to room temperature (25 °C). Finally, the parylene films were peeled from the supporting glasses. When hole patterns were required in the parylene films, the necessary areas were opened before the bonding process by oxygen plasma etching with a 12 sccm oxygen flow at 300 W for 15 min (PC‐300; SAMCO Inc., JPN). The necessary areas were defined by polyimide masks placed on parylene films during plasma etching. The polyimide masks were prepared through laser cutting (T‐Centric Laser Marker, MD‐T1000W; KEYENCE Co. Ltd., JPN). In the fabrication of OECTs, one pair of parylene films contained AgNW‐PEDOT:PSS composite electrodes for the source and drain as well as PEDOT:PSS for the channel, whereas the other endowed the hole patterns for ionic transport from the electrolyte to the channel. The structure profiles of the parylene films and PEDOT:PSS layer were measured using a stylus profilometer (DektakXT; Bruker corporation, USA).

### Fabrication of OECTs

The source, drain electrodes, and active channels were patterned via a rod‐coating process using hydrophilic‐/hydrophobic‐patterned surfaces in accordance with previous reports.^[^ ^31^, ^32^ ^]^ The hydrophilic‐/hydrophobic‐patterned surfaces were prepared on 1 µm parylene substrates by fluorocarbon coating (WP‐100; DAIKIN INDUSTRIES, Ltd., JPN) and vacuum‐ultraviolet (VUV) light irradiation through a photomask for 1 min. The samples were placed at a distance of 5 mm from the VUV lamp surface (Flat Excimer EX‐mini; Hamamatsu Photonics K.K., JPN). Then, for patterning the source and drain electrodes, AgNW dispersion was swept over the hydrophilic‐/hydrophobic‐patterned surfaces at 0.4 mm s^−1^ eight times in different directions, that is, parallel and perpendicular to the channel four times each, respectively, which enabled the patterning of cross‐aligned AgNW electrodes as the source and drain electrodes. For patterning the active channels, VUV light was irradiated through another photomask on the areas between the source and drain electrodes, that is, the prescribed channel areas, in a lamp setup similar to the source and drain electrodes. PEDOT:PSS dispersion was swept over the samples at 1.6 mm s^−1^ once in a direction parallel to the channel. The samples were heated at 120 °C in near‐vacuum condition (≈100 Pa) for 1 h. Finally, another parylene film with hole patterns was bonded to the samples containing the AgNW‐PEDOT:PSS source, drain electrodes, and PEDOT:PSS channels using the aforementioned bonding process.

### Electrical and Optical Characterization

The electrical characterization of OECTs was performed using a semiconductor parameter analyzer (B1500A; Keysight Technologies, USA). A gate voltage was applied to an Ag/AgCl electrode partially dipped into a droplet of saline solution placed on the channel holes. The optical observations of the samples were examined with an optical microscope (DM4000; Leica Microsystems GmbH, Germany) in a bright or dark field. The visible transmittance was measured using a microscopic spectrophotometer (MSV‐5000; JASCO Corporation, JPN). The frequency response of OECTs was measured using a source measurement unit system (B2962A; Keysight Technologies, USA). The optoelectronic performance of OECTs, that is, the transconductance and visible transmittance, was benchmarked against previously reported transparent transistors, as shown in Figure 2b. The transparent transistors were classified considering the channel materials, such as metal oxides (tin oxide, indium tin oxide, and indium gallium zinc oxide),^[^ ^48^, ^49^, ^50^, ^51^, ^52^, ^53^ ^]^ graphene and carbon nanotubes,^[^ ^50^, ^54^, ^55^, ^56^, ^57^, ^58^, ^59^, ^60^ ^]^ and conducting polymers.^[^ ^26^, ^28^, ^61^ ^]^ In the literature related to normalized transconductance based on the measured voltage, the transconductance was calculated by multiplying the normalized value with the drain or gate voltage. The transmittance of source/drain electrodes at a wavelength of 550 nm was used as that of the plot. If the transmittance spectra or measured points were not apparent, the transmittance denoted in the manuscript was extracted.

### Mechanical Characterization

All mechanical tests were performed using a compact tabletop tensile tester (EZ‐SX; Shimadzu Corporation, JPN). For the adhesion test of parylene bonding, the interfacial shear strength was measured with an overlap area (≈1 × 2 cm^2^). A pair of polyethylene terephthalate (PET) substrates coated with a 1 µm parylene layer was prepared via CVD. The adhesion strength between PET and parylene was strong enough for the adhesion test of parylene bonding. The pairs of parylene films or parylene‐coated PET substrates were bonded using the aforementioned process. Both ends of the bonded samples, which were unbonded single films, were gripped with pneumatic flat grips, and the tests were performed at a tensile speed of 0.1 mm s^−1^.

### EEG Acquisition and LDF

The acquisition setup is illustrated in Figure 5a. The source/drain electrodes were serially connected to registers to translate the current modulation into voltage signals. A bias of 1.5 V was applied using a commercial battery over the resistors and source/drain electrodes. The voltage measurements were implemented at a sampling rate of 250 Hz using a wireless module used in previous studies.^[^ ^32^, ^62^ ^]^ The wireless module equipped with a pin connector for each channel was connected to the terminal on a breadboard circuit based on Figure 5a via a metallic wire of ≈0.5 mm in diameter. A contact pad for the OECT drain was connected to a metallic wire (of the same diameter) using room temperature curable silver paste (TK paste; Kaken Tech Co., Ltd., Japan). The other end of the metal wire was connected to an Ag‐based plate electrode (1 cm in diameter) that was used with a conductive paste (with 12.5% NaCl content, used as a wet electrode, TEN20; Weaver and Co., USA) to attach near the mastoid process as a ground electrode. An OECT channel was directly placed on the forehead.^[^ ^47^ ^]^ The skin surface became a substantial gate. As for the source electrode side, after processing both ends of a metallic wire as described above, the contact pads of OECT and the breadboard circuit were connected. The acquired signals were processed using a band‐pass filter of 1–50 Hz. The spectrograms were obtained through wavelet transformation (Morlet wavelet) in a commercial software (Origin; OriginLab Corporation, USA). The blood flow was monitored using a laser Doppler blood flowmeter (ATBF II‐LC1; UNIQUE MEDICAL Co., Ltd., JPN). An optical probe was fixed on fully transparent ultrathin OECTs by sealing with adhesion tape (Tegaderm; 3M Company, USA). All biological signal measurement methods were approved by the Osaka University Research Ethics Committee and complied with the research guidelines of Osaka University (approval number: 31–2).

### Nitrate Ion Sensing

Figure 6a illustrates the measurement setup. For sensing with OECTs, a source measurement unit (B2912A; Keysight Technologies, USA) was used. The drain and gate voltages (*V*_DS_ and *V*_GS_) were constantly maintained at −0.8 and 0 V, respectively, while the drain current (*I*_DS_) was measured by increasing the nitrate ion concentration in deionized water or saline solution (15 mm NaCl aq.). A nitrate‐selective electrode^[^ ^44^ ^]^ was used for gate voltage application.

## Conflict of Interest

The authors declare no conflict of interest.

## Author Contributions

*Responsible for the concept, experiments, and structure*: A.T., T.A., S.W., J.M.J.T., and T.S. *Developed the fabrication process for OECTs*: A.T., T.A., and J.M.K. *Fabricated and characterized OECTs using the measurement setup optimized by T.U., M.A., T.S., T.A., T.U, and T.S*: A.T., J.M.K., K.N., K.K., and N.K. *Contributed to the nitrate ion sensing system*: T.A., Y.K., S.O., and S.W. The manuscript has been reviewed by all authors.

## Supporting information

Supporting Information

## Data Availability

Research data are not shared.

## References

[1] G.‐H. Lee , H. Moon , H. Kim , G. H. Lee , W. Kwon , S. Yoo , D. Myung , S. H. Yun , Z. Bao , S. K. Hahn , Nat. Rev. Mater. 2020, 5, 149. 32728478 10.1038/s41578-019-0167-3 PMC7388681

[2] C.‐C. Kim , H.‐H. Lee , K. H. Oh , J.‐Y. Sun , Science 2016, 353, 682. 27516597 10.1126/science.aaf8810

[3] S. Lim , D. Son , J. Kim , Y. B. Lee , J.‐K. Song , S. Choi , D. J. Lee , J. H. Kim , M. Lee , T. Hyeon , D.‐H. Kim , Adv. Funct. Mater. 2015, 25, 375.

[4] X. Pu , H. Guo , J. Chen , X. Wang , Y. Xi , C. Hu , Z. L. Wang , Sci. Adv. 2017, 3, e1700694. 28782029 10.1126/sciadv.1700694 PMC5533541

[5] D. Kuzum , H. Takano , E. Shim , J. C. Reed , H. Juul , A. G. Richardson , J. de Vries , H. Bink , M. A. Dichter , T. H. Lucas , D. A. Coulter , E. Cubukcu , B. Litt , Nat. Commun. 2014, 5, 5259. 25327632 10.1038/ncomms6259 PMC4331185

[6] D.‐W. Park , A. A. Schendel , S. Mikael , S. K. Brodnick , T. J. Richner , J. P. Ness , M. R. Hayat , F. Atry , S. T. Frye , R. Pashaie , S. Thongpang , Z. Ma , J. C. Williams , Nat. Commun. 2014, 5, 5258. 25327513 10.1038/ncomms6258 PMC4218963

[7] C. Lu , S. Park , T. J. Richner , A. Derry , I. Brown , C. Hou , S. Rao , J. Kang , C. T. Moritz , Y. Fink , P. Anikeeva , Sci. Adv. 2017, 3, e1600955. 28435858 10.1126/sciadv.1600955 PMC5371423

[8] J. Zhang , X. Liu , W. Xu , W. Luo , M. Li , F. Chu , L. Xu , A. Cao , J. Guan , S. Tang , X. Duan , Nano Lett. 2018, 18, 2903. 29608857 10.1021/acs.nanolett.8b00087

[9] N. Oh , B. H. Kim , S.‐Y. Cho , S. Nam , S. P. Rogers , Y. Jiang , J. C. Flanagan , Y. Zhai , J.‐H. Kim , J. Lee , Y. Yu , Y. K. Cho , G. Hur , J. Zhang , P. Trefonas , J. A. Rogers , M. Shim , Science 2017, 355, 616. 28183975 10.1126/science.aal2038

[10] H.‐H. Chou , A. Nguyen , A. Chortos , J. W. F. To , C. Lu , J. Mei , T. Kurosawa , W.‐G. Bae , J. B. H. Tok , Z. Bao , Nat. Commun. 2015, 6, 8011. 26300307 10.1038/ncomms9011 PMC4560774

[11] C. Wang , D. Hwang , Z. Yu , K. Takei , J. Park , T. Chen , B. Ma , A. Javey , Nat. Mater. 2013, 12, 899. 23872732 10.1038/nmat3711

[12] B. Lee , J.‐Y. Oh , H. Cho , C. W. Joo , H. Yoon , S. Jeong , E. Oh , J. Byun , H. Kim , S. Lee , J. Seo , C. W. Park , S. Choi , N.‐M. Park , S.‐Y. Kang , C.‐S. Hwang , S.‐D. Ahn , J.‐I. Lee , Y. Hong , Nat. Commun. 2020, 11, 663. 32005935 10.1038/s41467-020-14485-9 PMC6994701

[13] J. Kim , M. Kim , M.‐S. Lee , K. Kim , S. Ji , Y.‐T. Kim , J. Park , K. Na , K.‐H. Bae , H. Kyun Kim , F. Bien , C. Young Lee , J.‐U. Park , Nat. Commun. 2017, 8, 14997. 28447604 10.1038/ncomms14997 PMC5414034

[14] J. Park , J. Kim , S.‐Y. Kim , W. H. Cheong , J. Jang , Y.‐G. Park , K. Na , Y.‐T. Kim , J. H. Heo , C. Y. Lee , J. H. Lee , F. Bien , J.‐U. Park , Sci. Adv. 2018, 4, eaap9841. 29387797 10.1126/sciadv.aap9841 PMC5787380

[15] G. W. Gross , W. Y. Wen , J. W. Lin , J. Neurosci. Methods 1985, 15, 243. 4094480 10.1016/0165-0270(85)90105-0

[16] C. Pan , L. Dong , G. Zhu , S. Niu , R. Yu , Q. Yang , Y. Liu , Z. L. Wang , Nat. Photonics 2013, 7, 752.

[17] F. Al‐Shargie , M. Kiguchi , N. Badruddin , S. C. Dass , A. F. M. Hani , T. B. Tang , Biomed. Opt. Express 2016, 7, 3882. 27867700 10.1364/BOE.7.003882 PMC5102531

[18] J. Wang , H. Rao , G. S. Wetmore , P. M. Furlan , M. Korczykowski , D. F. Dinges , J. A. Detre , Proc. Natl. Acad. Sci. U. S. A. 2005, 102, 17804. 16306271 10.1073/pnas.0503082102 PMC1292988

[19] T. Sannicolo , M. Lagrange , A. Cabos , C. Celle , J.‐P. Simonato , D. Bellet , Small 2016, 12, 6052. 27753213 10.1002/smll.201602581

[20] L. Yu , C. Shearer , J. Shapter , Chem. Rev. 2016, 116, 13413. 27704787 10.1021/acs.chemrev.6b00179

[21] H. Jang , Y. J. Park , X. Chen , T. Das , M.‐S. Kim , J.‐H. Ahn , Adv. Mater. 2016, 28, 4184. 26728114 10.1002/adma.201504245

[22] J. Rivnay , S. Inal , A. Salleo , R. M. Owens , M. Berggren , G. G. Malliaras , Nat. Rev. Mater. 2018, 3, 17086.

[23] D. Khodagholy , J. Rivnay , M. Sessolo , M. Gurfinkel , P. Leleux , L. H. Jimison , E. Stavrinidou , T. Herve , S. Sanaur , R. M. Owens , G. G. Malliaras , Nat. Commun. 2013, 4, 2133. 23851620 10.1038/ncomms3133 PMC3717497

[24] M. Sessolo , D. Khodagholy , J. Rivnay , F. Maddalena , M. Gleyzes , E. Steidl , B. Buisson , G. G. Malliaras , Adv. Mater. 2013, 25, 2135. 23417987 10.1002/adma.201204322

[25] D. Khodagholy , T. Doublet , P. Quilichini , M. Gurfinkel , P. Leleux , A. Ghestem , E. Ismailova , T. Hervé , S. Sanaur , C. Bernard , G. G. Malliaras , Nat. Commun. 2013, 4, 1575. 23481383 10.1038/ncomms2573 PMC3615373

[26] W. Lee , D. Kim , N. Matsuhisa , M. Nagase , M. Sekino , G. G. Malliaras , T. Yokota , T. Someya , Proc. Natl. Acad. Sci. U. S. A. 2017, 114, 10554. 28923928 10.1073/pnas.1703886114 PMC5635873

[27] S. Park , S. W. Heo , W. Lee , D. Inoue , Z. Jiang , K. Yu , H. Jinno , D. Hashizume , M. Sekino , T. Yokota , K. Fukuda , K. Tajima , T. Someya , Nature 2018, 561, 516. 30258137 10.1038/s41586-018-0536-x

[28] C. Cea , G. D. Spyropoulos , P. Jastrzebska‐Perfect , J. J. Ferrero , J. N. Gelinas , D. Khodagholy , Nat. Mater. 2020, 19, 679. 32203456 10.1038/s41563-020-0638-3

[29] D. Ohayon , G. Nikiforidis , A. Savva , A. Giugni , S. Wustoni , T. Palanisamy , X. Chen , I. P. Maria , E. Di Fabrizio , P. M. F. J. Costa , I. McCulloch , S. Inal , Nat. Mater. 2020, 19, 456. 31844278 10.1038/s41563-019-0556-4

[30] M. Kaltenbrunner , T. Sekitani , J. Reeder , T. Yokota , K. Kuribara , T. Tokuhara , M. Drack , R. Schwödiauer , I. Graz , S. Bauer‐Gogonea , S. Bauer , T. Someya , Nature 2013, 499, 458. 23887430 10.1038/nature12314

[31] A. Takemoto , T. Araki , Y. Noda , T. Uemura , S. Yoshimoto , R. Abbel , C. Rentrop , J. van den Brand , T. Sekitani , Nanotechnology 2019, 30, 37LT03. 10.1088/1361-6528/ab2aad 31212258

[32] A. Takemoto , T. Araki , T. Uemura , Y. Noda , S. Yoshimoto , S. Izumi , S. Tsuruta , T. Sekitani , Adv Intell. Syst. 2020, 2, 2000093.

[33] L. Yang , T. Zhang , H. Zhou , S. C. Price , B. J. Wiley , W. You , ACS Appl. Mater. Interfaces 2011, 3, 4075. 21899278 10.1021/am2009585

[34] D. A. Bernards , G. G. Malliaras , Adv. Funct. Mater. 2007, 17, 3538.

[35] J. T. Friedlein , M. J. Donahue , S. E. Shaheen , G. G. Malliaras , R. R. McLeod , Adv. Mater. 2016, 28, 8398. 27457055 10.1002/adma.201602684

[36] A. J. Casson , D. C. Yates , S. J. M. Smith , J. S. Duncan , E. Rodriguez‐Villegas , IEEE Eng. Med. Biol. Mag. 2010, 29, 44. 10.1109/MEMB.2010.936545 20659857

[37] W. Klimesch , Brain Res. Rev. 1999, 29, 169. 10209231 10.1016/s0165-0173(98)00056-3

[38] K.‐I. Jang , S. Y. Han , S. Xu , K. E. Mathewson , Y. Zhang , J.‐W. Jeong , G.‐T. Kim , R. C. Webb , J. W. Lee , T. J. Dawidczyk , R. H. Kim , Y. M. Song , W.‐H. Yeo , S. Kim , H. Cheng , S. Il Rhee , J. Chung , B. Kim , H. U. Chung , D. Lee , Y. Yang , M. Cho , J. G. Gaspar , R. Carbonari , M. Fabiani , G. Gratton , Y. Huang , J. A. Rogers , Nat. Commun. 2014, 5, 4779. 25182939 10.1038/ncomms5779

[39] N. Z. Gurel , H. Jung , S. Hersek , O. T. Inan , IEEE Sens. J. 2019, 19, 8522. 33312073 10.1109/jsen.2018.2872651 PMC7731966

[40] E. R. J. Seitsonen , I. K. J. Korhonen , M. J. van Gils , M. Huiku , J. M. P. Lötjönen , K. T. Korttila , A. M. Yli‐Hankala , Acta Anaesthesiol. Scand. 2005, 49, 284. 15752389 10.1111/j.1399-6576.2005.00654.x

[41] S. Fazli , J. Mehnert , J. Steinbrink , G. Curio , A. Villringer , K. R. Müller , B. Blankertz , NeuroImage 2012, 59, 519. 21840399 10.1016/j.neuroimage.2011.07.084

[42] K. Kitamura , K. Murai , S. Wakida , T. Miyado , K. Fukushi , Y. Hayashi , IEEJ Trans. Electr. Electron. Eng. 2013, 8, 301.

[43] L. Jin , L. Qin , D. Xia , X. Liu , Z. Fan , C. Zhang , L. Gu , J. He , I. S. Ambudkar , D. Deng , S. Wang , F. Radic , Biol. Med. 2013, 57, 61. 10.1016/j.freeradbiomed.2012.12.015 PMC4059197 23277147

[44] S. Osaki , T. Kintoki , T. Moriuchi‐Kawakami , K. Kitamura , S. Wakida , Sensors 2019, 19, 2713. 31212895 10.3390/s19122713 PMC6630929

[45] J. O. Lundberg , E. Weitzberg , J. A. Cole , N. Benjamin , Nat. Rev. Microbiol. 2004, 2, 593. 15197394 10.1038/nrmicro929

[46] H. Kim , K. Najafi , J. Microelectromech. Syst. 2005, 14, 1347.

[47] T. Araki , S. Yoshimoto , T. Uemura , A. Miyazaki , N. Kurihira , Y. Kasai , Y. Harada , T. Nezu , H. Iida , J. Sandbrook , S. Izumi , T. Sekitani , Adv. Mater. Technol. 2022, 2200362.

[48] S. Li , M. Tian , Q. Gao , M. Wang , T. Li , Q. Hu , X. Li , Y. Wu , Nat. Mater. 2019, 18, 1091. 31406368 10.1038/s41563-019-0455-8

[49] E. N. Dattoli , Q. Wan , W. Guo , Y. Chen , X. Pan , W. Lu , Nano Lett. 2007, 7, 2463. 17595151 10.1021/nl0712217

[50] H. Chen , Y. Cao , J. Zhang , C. Zhou , Nat. Commun. 2014, 5, 4097. 24923382 10.1038/ncomms5097

[51] G. A. Salvatore , N. Münzenrieder , T. Kinkeldei , L. Petti , C. Zysset , I. Strebel , L. Büthe , G. Tröster , Nat. Commun. 2014, 5, 2982. 24399363 10.1038/ncomms3982

[52] S. Park , S. Lee , C.‐H. Kim , I. Lee , W.‐J. Lee , S. Kim , B.‐G. Lee , J.‐H. Jang , M.‐H. Yoon , Sci. Rep. 2015, 5, 13088. 26271456 10.1038/srep13088 PMC4642510

[53] I. Cunha , R. Barras , P. Grey , D. Gaspar , E. Fortunato , R. Martins , L. Pereira , Adv. Funct. Mater. 2017, 27, 1606755.

[54] J. U. Park , S. Nam , M. S. Lee , C. M. Lieber , Nat. Mater. 2012, 11, 120. 10.1038/nmat3169 PMC3602909 22101813

[55] L. Liao , Y.‐C. Lin , M. Bao , R. Cheng , J. Bai , Y. Liu , Y. Qu , K. L. Wang , Y. Huang , X. Duan , Nature 2010, 467, 305. 20811365 10.1038/nature09405 PMC2965636

[56] F. N. Ishikawa , H. Chang , K. Ryu , P. Chen , A. Badmaev , L. Gomez De Arco , G. Shen , C. Zhou , ACS Nano 2009, 3, 73. 19206251 10.1021/nn800434d

[57] J. Zhang , C. Wang , C. Zhou , ACS Nano 2012, 6, 7412. 22788112 10.1021/nn3026172

[58] S. Kim , S. Ju , J. H. Back , Y. Xuan , P. D. Ye , M. Shim , D. B. Janes , S. Mohammadi , Adv. Mater. 2009, 21, 564. 21161982 10.1002/adma.200801032

[59] J. Liang , L. Li , D. Chen , T. Hajagos , Z. Ren , S.‐Y. Chou , W. Hu , Q. Pei , Nat. Commun. 2015, 6, 7647. 26173436 10.1038/ncomms8647 PMC4518309

[60] S. H. Chae , W. J. Yu , J. J. Bae , D. L. Duong , D. Perello , H. Y. Jeong , Q. H. Ta , T. H. Ly , Q. A. Vu , M. Yun , X. Duan , Y. H. Lee , Nat. Mater. 2013, 12, 403. 23455851 10.1038/nmat3572

[61] G. D. Spyropoulos , J. N. Gelinas , D. Khodagholy , Sci. Adv. 2019, 5, eaau7378. 30820453 10.1126/sciadv.aau7378 PMC6392764

[62] T. Araki , T. Uemura , S. Yoshimoto , A. Takemoto , Y. Noda , S. Izumi , T. Sekitani , Adv. Mater. 2020, 32, 1902684. 10.1002/adma.201902684 31782576

