# Supplementary material for: Fully Transparent, Ultrathin Flexible Organic Electrochemical Transistors with Additive Integration for Bioelectronic Applications
Source: Adv Sci (Weinh). 2022 Nov 14;10(2):2204746. doi: 10.1002/advs.202204746 (PMC9839865; doi:10.1002/advs.202204746)
Supplement: Supplementary file 1 — Supporting Information [file ADVS-10-2204746-s001.pdf]

ADVANCED SCIENCE
Open Access

Supporting Information

for *Adv. Sci.*, DOI 10.1002/advs.202204746

Fully Transparent, Ultrathin Flexible Organic Electrochemical Transistors with Additive Integration for Bioelectronic Applications

Ashuya Takemoto, Teppei Araki, Kazuya Nishimura, Mihoko Akiyama, Takafumi Uemura, Kazuki Kiriyama, Johan M. Koot, Yuko Kasai, Naoko Kurihira, Shuto Osaki, Shin-ichi Wakida, Jaap M.J. den Toonder and Tsuyoshi Sekitani**

Supplementary Information

Fully Transparent, Ultrathin Flexible Organic Electrochemical Transistors with Additive Integration for Bioelectronic Applications

Ashuya Takemoto[1,2,3], Teppei Araki[1,2,3], Kazuya Nishimura [1,2,3], Mihoko Akiyama[1], Takafumi Uemura[1,3], Kazuki Kiriyama[1,2,3], Johan M. Koot[4], Yuko Kasai[3], Naoko Kurihira[1], Shuto Osaki[2,3], Shin-ichi Wakida[2,3], Jaap M.J. den Toonder[4], Tsuyoshi Sekitani*[1,2,3]*

[1]The Institute of Scientific and Industrial Research (SANKEN), Osaka University, Ibaraki, 567-0047, Japan

[2]Department of Applied Physics, Graduate School of Engineering, Osaka University, Suita, 565-0871, Japan

[3]Advanced Photonics and Biosensing Open Innovation Laboratory, AIST-Osaka University, Suita, 565-0871, Japan

[4]Department of Mechanical Engineering and Institute for Complex Molecular Systems, Eindhoven University of Technology, Eindhoven, 5600 MB, The Netherlands

Structure and Adhesion Performance of Thermal Lamination

The encapsulation layer with openings on the channel is a key component in OECTs. It offers ion-injection/ejection paths between the channel and electrolytes and insulates the source/drain from the electrolytes. In previous studies on flexible OECTs, the encapsulation layers were prepared by depositing hole-less layers, followed by the reactive-ion etching process to pattern the openings.[1,2] In contrast, we introduced an additive integration without the etching process, i.e., the thermal lamination method[3,4] (**Figures S1 and S2**). The encapsulation layer can be additively deposited with hole patterns corresponding to channel openings. The lamination mechanism was based on the entanglement of polymer chains during heat pressing.[3,4] This process permits layer formation without deteriorative effects (decomposition or oxidization) on PEDOT:PSS channels and AgNW electrodes, while the previously used etching process for the openings can expose the active channels or source/drain electrodes of OECTs to reactive ions or harsh chemicals.

Moreover, it is important to retain the form factors of constituent materials for the development of OECTs. Remarkably, in this method, the structures of the encapsulation layer and base film were retained. **Figure S3** shows a step profile of the laminated and base films, which approximately have the same thickness (1 μm) as before lamination. Therefore, the source/drain electrodes can be easily placed in a neutral strain position between parylene films of the same thickness, which is favorable for mechanical stability.[5] Additionally, various opening designs with a width of up to 20 μm can be retained. For example, parylene films with holes of size 50 μm (source/drain design), cross-patterned holes (alignment marks), and boundary lines were successfully laminated as encapsulation layers on the base parylene films (**Figures S3** and **S4**). The designability of hole patterns in the encapsulation layer enabled us to pattern the openings of the channels, gate electrodes, and source/drain contacts.

The adhesion of the encapsulation layer in flexible devices is critical for mechanical stability. To evaluate the adhesion between the parylene substrate and encapsulation layer, the shear bond strength was measured through tensile tests (**Figure S5a, b**). We used a polyethylene terephthalate (PET) film to support a 1 μm parylene layer. The PET films effectively prevented the

delamination of parylene layers from the supporting films during the tensile tests. Furthermore, we coated the parylene substrate with fluorocarbon agents, which are required for selective wetting deposition. **Figure S5b** shows that the shear strength of 287±64 kPa for the parylene-bonded interfaces with intermediate fluorocarbons was comparable to that without fluorocarbons (307±36 kPa). The minimal effect of intermediate fluorocarbons on the shear strength may be ascribed to the penetration of parylene polymer chains by fluorocarbons with a thickness of a few nanometers. Next, we investigated whether the adhesion is high enough for ultrathin devices by comparing two fracture forces (i.e., tensile fracture in parylene films and interface shear fracture) estimated from the tensile strength of parylene film (**Figure S5c**) and shear bond strength. The shear fracture force was more than 100 times higher than the tensile fracture force, as shown in **Figure S5d**. The results indicate that the adhesion was sufficiently high. Furthermore, we confirmed that the unbonded parts of parylene films were fractured, while the bonded parts remained intact when the free-standing parylene films were bonded and used for measurements (**Figure S6**). Such strong bonding ensures that the encapsulation layer can be implemented without deteriorating the mechanical stability of ultrathin OECTs.

Optical Characterization of Fully Transparent Ultrathin OECTs

The high optical transparency of bioelectronic sensors is an attractive feature because optical assessments of biological organisms can be conducted directly above the sensors during an electrical assessment. The capability of multimodal assessments may advance the diagnostic quality of wearable monitoring systems. To evaluate the optical characterization of OECTs, the transmittance spectra in the visible range were obtained for specific device areas of encapsulation and substrate, source/drain electrodes, and channel, as shown in **Figures 2h** and **S7**. Each area on OECTs exhibits a high transmittance of approximately 90% in the visible range of 400−800 nm (**Figure S7b**). **Figures 2h** and **S7c** show the visible transmittance (~97%) of the source/drain electrodes and channel, excluding the encapsulation and substrate for investigating the material properties, which are higher than those of previously reported transparent transistors (**Figure 2b**).

Current Gain in Frequency Characterization

The cutoff frequency of OECTs was determined as the frequency at which the current gain ($\Delta I_{DS}/\Delta I_{GS}$ and I_{DS} and I_{GS} denote the drain and gate currents, respectively.) becomes 1. The evaluation of cutoff frequency based on a current gain of 1 is essential for the frequency characterization of transistors because it represents the maximum frequency of input signals that are effectively amplified by transistors.[6] To characterize the frequency performance, first, as shown in **Figure S9a**, the frequency plots of the drain and gate currents were obtained to calculate the current gain at specific input signal frequencies. **Figure S9b** shows the representative current gain of OECTs with L/W = 50/200 µm as a function of the input frequency. The cutoff frequencies were then extracted by fitting a $1/f$ function to the current gain-frequency plots of OECTs with different channel lengths (L) ranging from 20–200 µm and a channel width of 200 µm. As shown in **Figure 3f**, the relationship between the extracted cutoff frequencies and channel length (L) was obtained. This demonstrates a good fit for the $1/L^2$ function, which is reasonable for the transistor theoretical equation of the cutoff frequency:[6]

$$f_{\text{cutoff}} = \frac{g_m}{2\pi\, C_G} = \frac{\mu}{2\pi\, L^2}(V_{GS}-V_{th}), \quad (1)$$

f_{cutoff}, g_m, and C_G denote the cutoff frequency, transconductance, and area-normalized capacitance, respectively; μ, V_{GS}, and V_{th} denote the mobility, gate voltage, and threshold gate voltage, respectively. The maximum cutoff frequency around 560 Hz was slower than other candidates for transparent transistors (i.e., metal oxides, carbon nanotubes, and graphene), as shown in **Figure 2b**; however, it was fast enough to record most biological signals because the signal frequency was less than 1 kHz.[7] Furthermore, the frequency performance may be improved by reducing the resistance of silver nanowire electrodes through conductive coating techniques, such as graphene layering,[8] gold plating,[9] or secondary doping of PEDOT:PSS[10].

Vertical Placement of Optical Sensors on OECTs

Multifunctional biomedical probes allow us to obtain versatile vital signals, leading to the extraction of more accurate information for medical diagnosis and brain–machine interfaces.[11] In particular, multifunctional probes combined with electrophysiological and optical sensors are among the most compelling candidates for wearable applications owing to their enhanced portability.[12] Thus far, the combination of EEG and functional near-infrared spectroscopy has demonstrated the ability to capture richer information related to human cortical activity.[12,13] The combined probes were devised for placing electrophysiological sensors next to optical sensors over the objected areas (forehead), as shown in **Figure S11a**. However, such in-plane placement of sensors inevitably reduced the spatial resolution in comparison to monofunctional probes because spacings between electrophysiological (optical) sensors must be required for one optical (electrophysiological) sensor. To circumvent this, we proposed a vertical placement enabled by transparent electrophysiological sensors, i.e., fully transparent ultrathin OECTs (**Figure S11b**). In such vertical placements, the optical sensors are placed directly above OECTs without spacing owing to high optical transmittance, which causes minimal interruptions in the optical path for sensing. **Figures S11c** and **S11d** show that the optical blood flow on the fingertip was measured to obtain the human pulse wave, even when the OECTs covered the fingertip. This demonstrates that the sensors can be stacked vertically without losing the functionality of optical sensing, considering their mutual alignment. Thus, the spatial resolution of electrophysiological and optical sensors can be enhanced to the same extent as that of monofunctional probes when the electrophysiological sensors are optically transparent and flexible.

References

[1] M. Sessolo, D. Khodagholy, J. Rivnay, F. Maddalena, M. Gleyzes, E. Steidl, B. Buisson, G. G. Malliaras, *Adv. Mater.* **2013**, *25*, 2135.

[2] D. Khodagholy, J. Rivnay, M. Sessolo, M. Gurfinkel, P. Leleux, L. H. Jimison, E. Stavrinidou, T. Herve, S. Sanaur, R. M. Owens, G. G. Malliaras, *Nat. Commun.* **2013**, *4*, 2133.

[3] H. Kim, K. Najafi, *J. Microelectromech. Syst.* **2005**, *14*, 1347.

[4] D. Ziegler, T. Suzuki, S. Takeuchi, *J. Microelectromech. Syst.* **2006**, *15*, 1477.

[5] Z. Suo, E. Y. Ma, H. Gleskova, S. Wagner, *Appl. Phys. Lett.* **1999**, *74*, 1177.

[6] S. M. Sze, M.-K. Lee, *Technology* **2002**, 568.

[7] P. Leleux, J. Rivnay, T. Lonjaret, J.-M. Badier, C. Bénar, T. Hervé, P. Chauvel, G. G. Malliaras, *Adv. Healthcare Mater.* **2015**, *4*, 142.

[8] I. N. Kholmanov, C. W. Magnuson, A. E. Aliev, H. Li, B. Zhang, J. W. Suk, L. L. Zhang, E. Peng, S. H. Mousavi, A. B. Khanikaev, R. Piner, G. Shvets, R. S. Ruoff, *Nano Lett.* **2012**, *12*, 5679.

[9] T. Araki, F. Yoshida, T. Uemura, Y. Noda, S. Yoshimoto, T. Kaiju, T. Suzuki, H. Hamanaka, K. Baba, H. Hayakawa, T. Yabumoto, H. Mochizuki, S. Kobayashi, M. Tanaka, M. Hirata, T. Sekitani, *Adv. Healthcare Mater.* **2019**, *8*, 1900130.

[10] X. Wu, A. Surendran, J. Ko, O. Filonik, E. M. Herzig, P. Müller-Buschbaum, W. L. Leong, *Adv. Mater.* **2019**, *31*, 1805544.

[11] L. Wang, Z. Lou, K. Jiang, G. Shen, *Adv. Intell. Syst.* **2019**, *1*, 1900040.

[12] F. Al-Shargie, T. B. Tang, M. Kiguchi, *Biomed. Opt. Express* **2017**, *8*, 2583.

[13] S. Fazli, J. Mehnert, J. Steinbrink, G. Curio, A. Villringer, K. R. Müller, B. Blankertz, *NeuroImage* **2012**, *59*, 519.

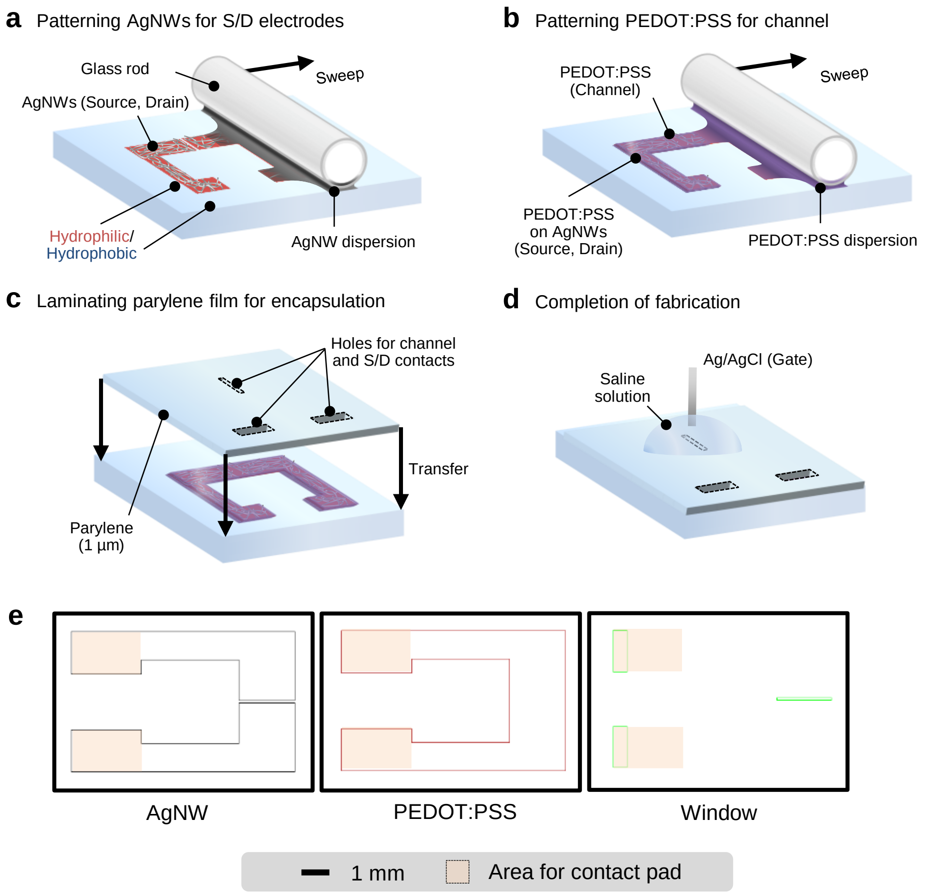

Figure S1. Fabrication process for ultrathin and transparent OECTs. **(a, b)** Patterning of AgNWs **(a)** and PEDOT:PSS **(b)** for source/drain electrodes and channel, respectively, via selective-wetting deposition. **(c)** Thermal lamination of parylene film with hole patterns for encapsulating the source/drain electrodes. **(d)** Completion of device fabrication. (**e**) Design of each layer when channel length and width are 100 and 2000 μm, respectively.

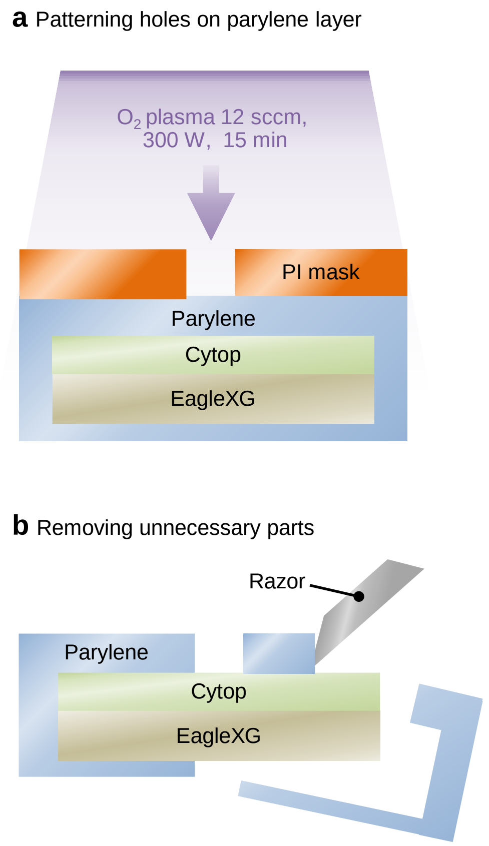

Figure S2. Thermal lamination for parylene films. **(a)** Patterning holes on a parylene film via O_2 plasma etching. **(b)** Removing unnecessary parts on the parylene film. **(c)** Transferring the parylene film with hole patterns for encapsulating a base using the thermal lamination method. **(d)** Completion of thermal lamination of the parylene film.

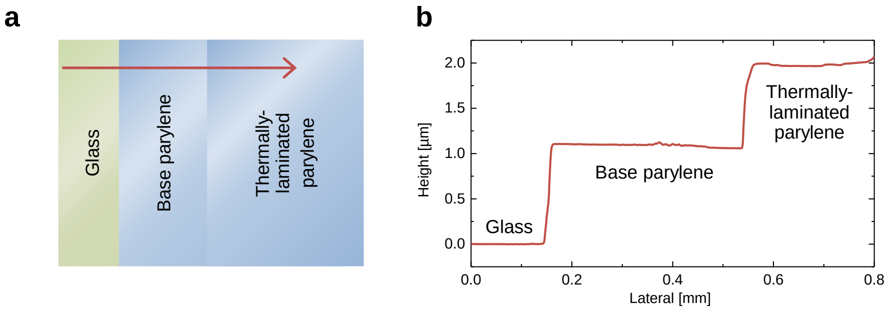

Figure S3. Configuration of the laminated parylene structure. **(a)** Schematic top view of the parylene structure composed of glass, base parylene, and thermally-laminated parylene film on the base. (**b**) Height profile of the structure corresponding to the red arrow in **a**.

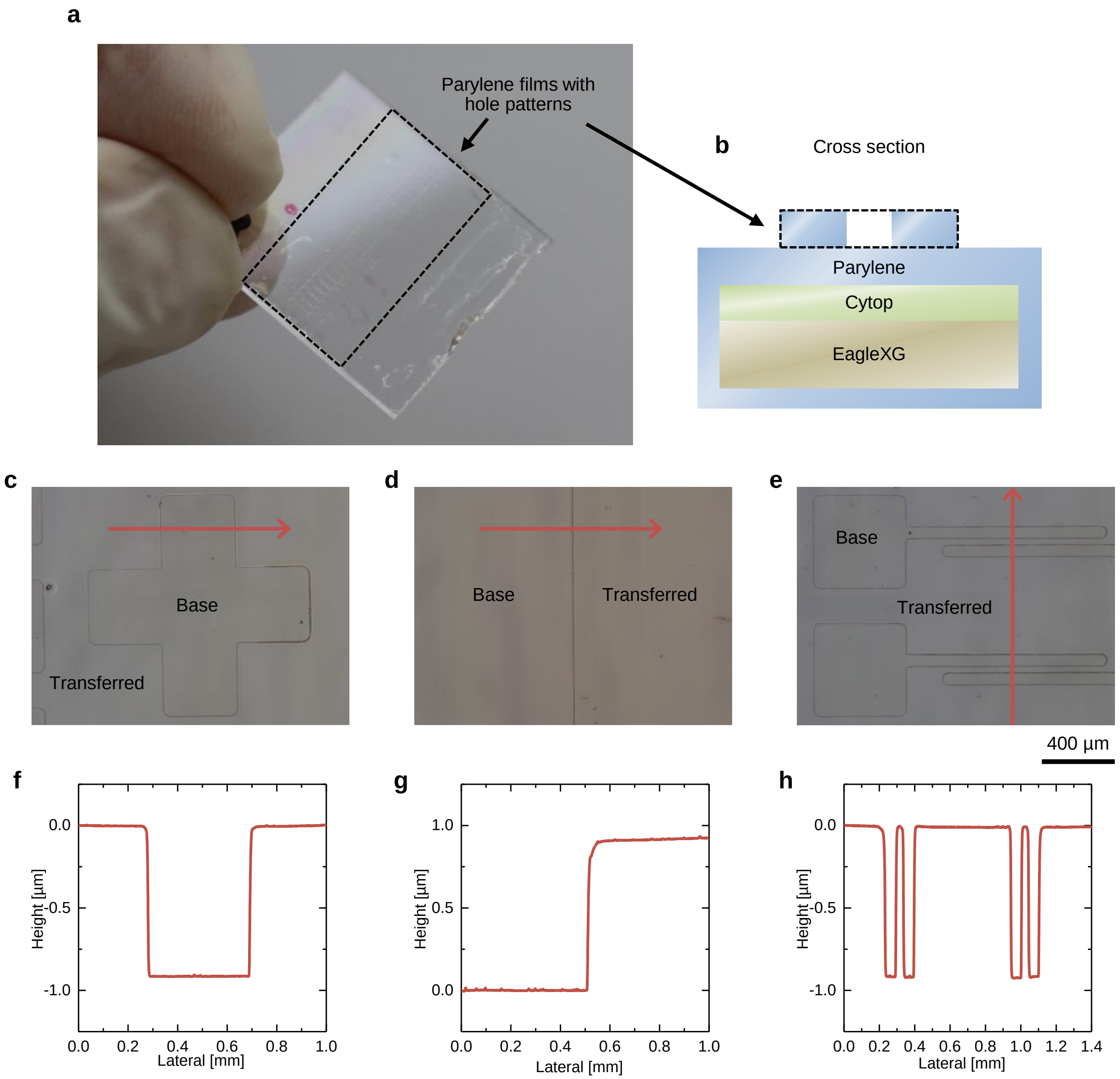

Figure S4. Thermally laminated parylene films. **(a, b)** Macroscopic photograph **(a)** and schematic **(b)** of parylene films supported on the glass substrate. **(c–e)** Optical micrographs of a cross-shaped hole **(c)**, borderline **(d)**, and source/drain electrode-shaped holes **(e)** on the base parylene films. **(f–h)** Height profiles corresponding to the red arrows in **c** (**f**), **d** (**g**), and **e** (**h**).

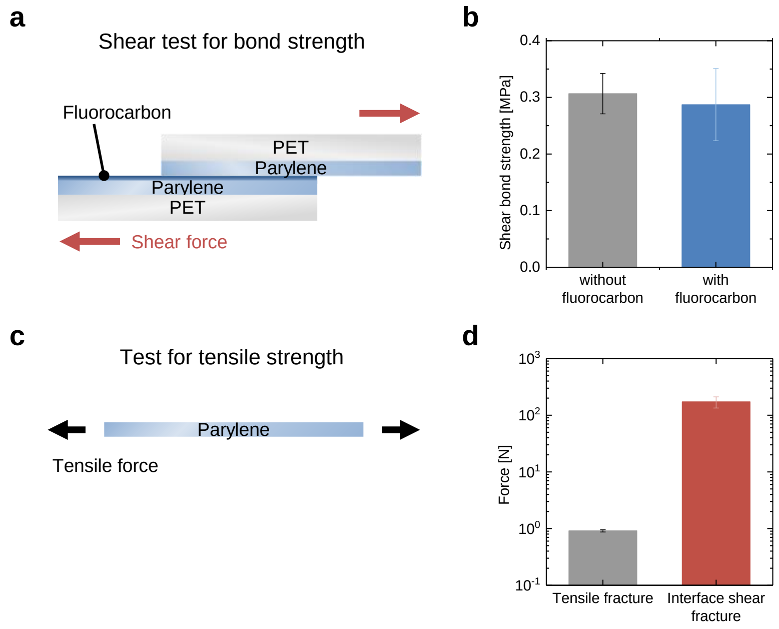

Figure S5. Adhesion performance of thermally laminated parylene films. **(a**, **b**) Schematic **(a)** of mechanical testing for bonded pairs of parylene films with and without an intermediate fluorocarbon layer and the resulting interface shear strength **(b)**. **(c)** Schematic of stretching the free-standing parylene films. **(d**) Comparison of fracture forces between the tensile fractures of parylene film and shear fracture of bonded parylene films. The forces were estimated with the effective device size (area = 2×3 cm^2, thickness = 1 μm).

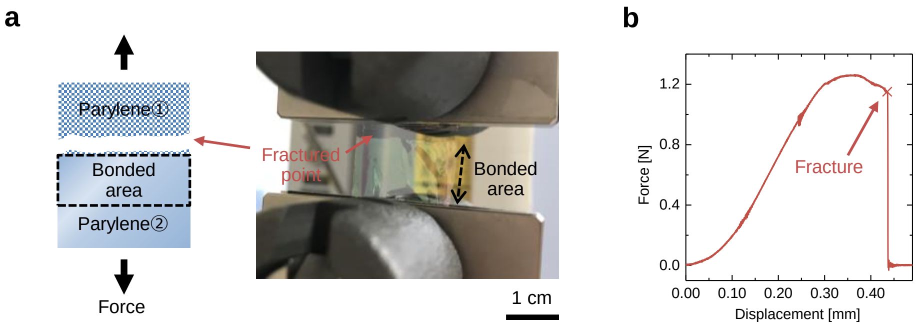

Figure S6. Mechanical test for adhesion performance of bonded pair of free-standing parylene films. **(a)** Schematic and photograph of fractured parylene film on the unbonded area. **(b)** Force-displacement curve measured with the bonded pair.

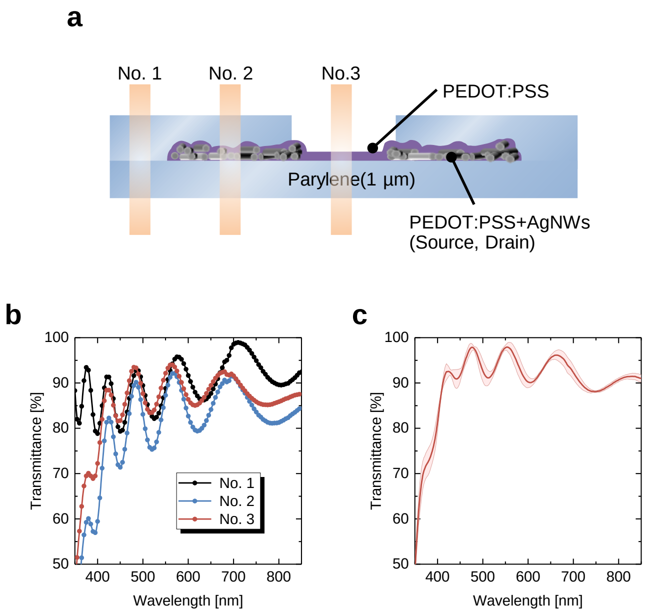

Figure S7. Optical characterization of fully transparent, ultrathin OECTs. **(a)** Schematic cross-section of OECTs with highlighted optical paths (No. 1–3) for optical transmittance measurement. **(b)** Visible transmittance spectra for 2 μm parylene films (encapsulation and substrate, No. 1), 1 μm parylene/AgNWs-PEDOT:PSS/1 μm parylene (source and drain, No. 2), and PEDOT:PSS/1 μm parylene (channel, No. 3). **(c)** Visible transmittance spectra of PEDOT:PSS without the substrate. The shaded region represents the standard deviation in 6 samples.

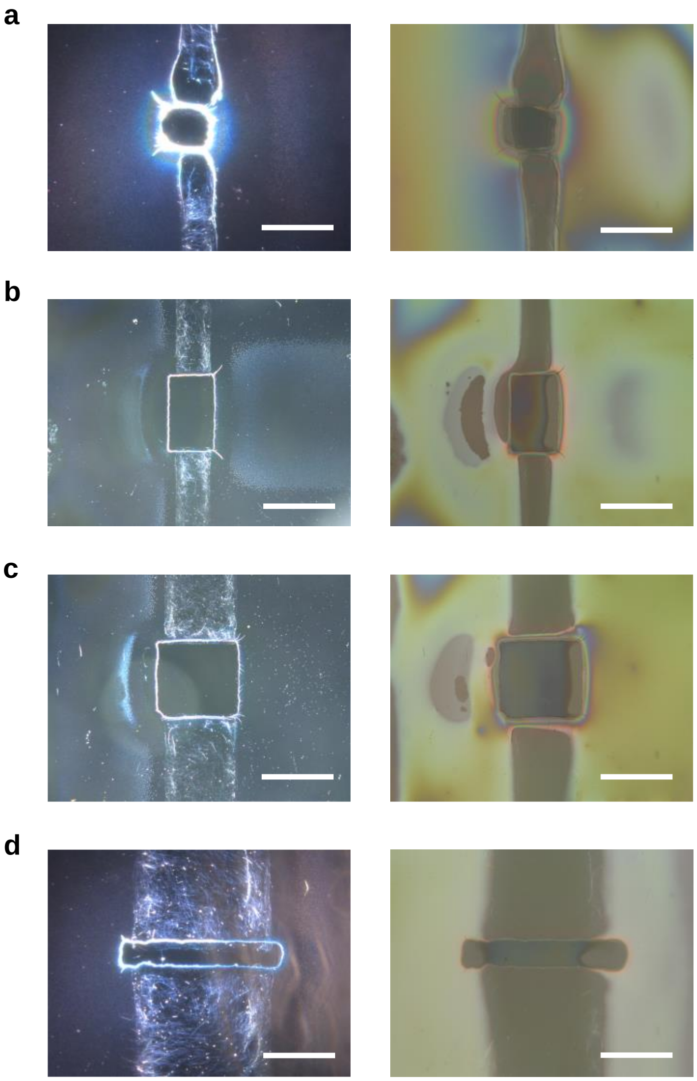

Figure S8. Scaled OECTs with different channel dimensional areas. **(a–d)** Optical micrographs of OECTs with channel width/length (*W*/*L*) = 50/50 μm **(a)**, *W*/*L* = 50/100 μm **(b)**, *W*/*L* = 100/100 μm **(c)**, and *W*/*L* = 200/20 μm **(d)**. The left and right micrographs correspond to dark-field and bright-field images, respectively. All scale bars correspond to 100 μm.

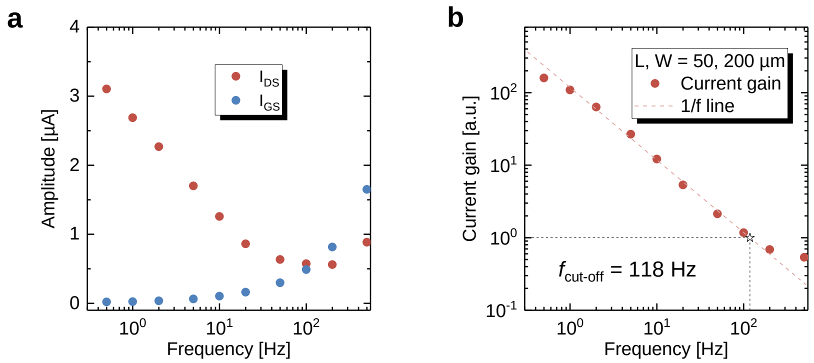

Figure S9. Extraction of the cutoff frequency of OECTs. **(a)** Amplitudes of drain current (red dots) and gate current (blue dots) of an OECT with *L*/*W* = 50/200 µm as a function of the frequency of the sinusoidal gate voltage with an amplitude of 0.01 V. **(b)** Current gain of OECTs as a function of frequency with 1/*f* fitting results (red dashed lines). The black dashed lines denote a gain of 1 and the extracted cutoff frequency.

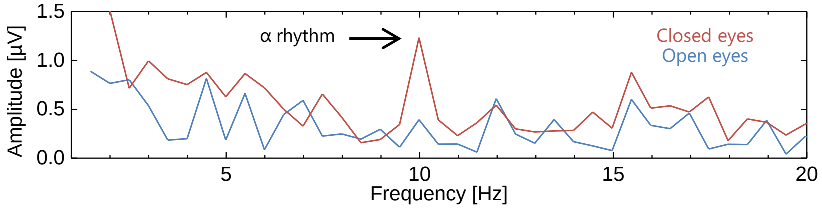

Figure S10. EEG acquisition using OECTs. Fast Fourier transform results for the acquired traces in **Figure 5b**. The red and blue lines correspond to the traces during closed and open eyes, respectively.

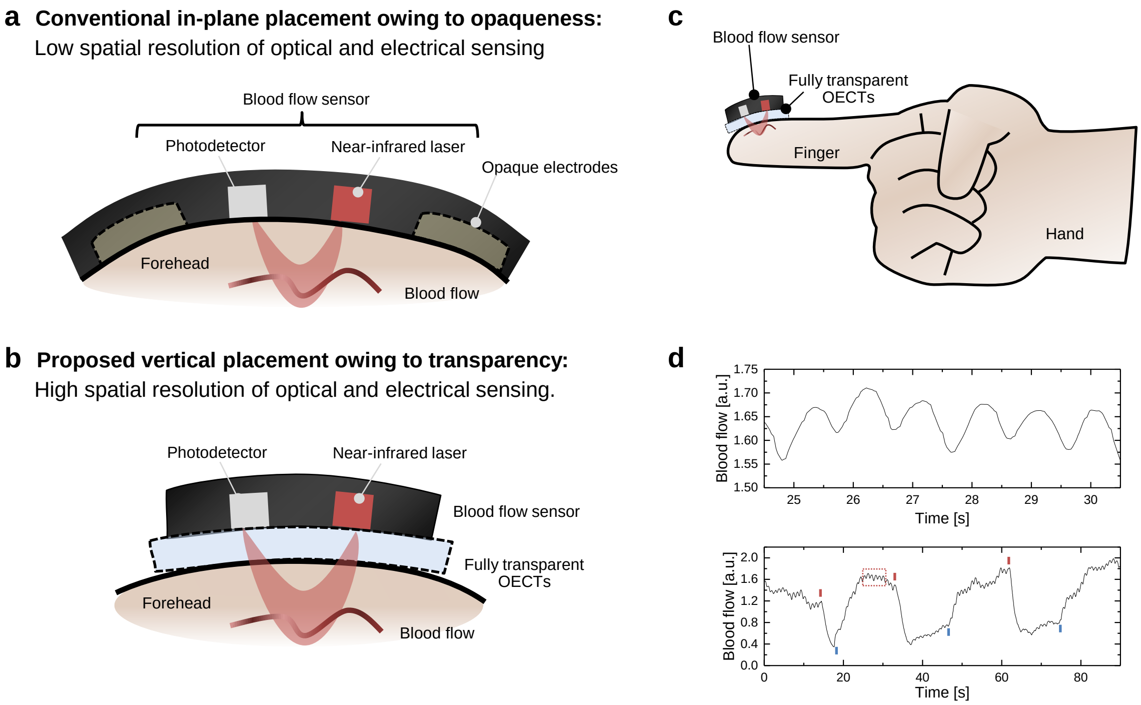

Figure S11. Capability for simultaneous LDF sensing. **(a, b)** Conceptual diagrams of advantages of fully transparent ultrathin OECTs. Optical transparency allows the vertical placement of optical and electrical sensors, unlike the conventional in-plane placement, which can be useful for high-resolution measurements of electrophysiological signals and optical assessments simultaneously. **(c)** Schematic of pulse wave measurements on a fingertip using LDF sensors placed directly above OECTs. **(d)** Time traces of pulse wave measurements. The upper part is a zoomed-in picture of the bottom time trace (red-dashed rectangle), showing individual pulses at 60 beats per minute. The red dots in the bottom time trace show the starting points of pressuring on the forearm, while the blue dots show the releasing points.

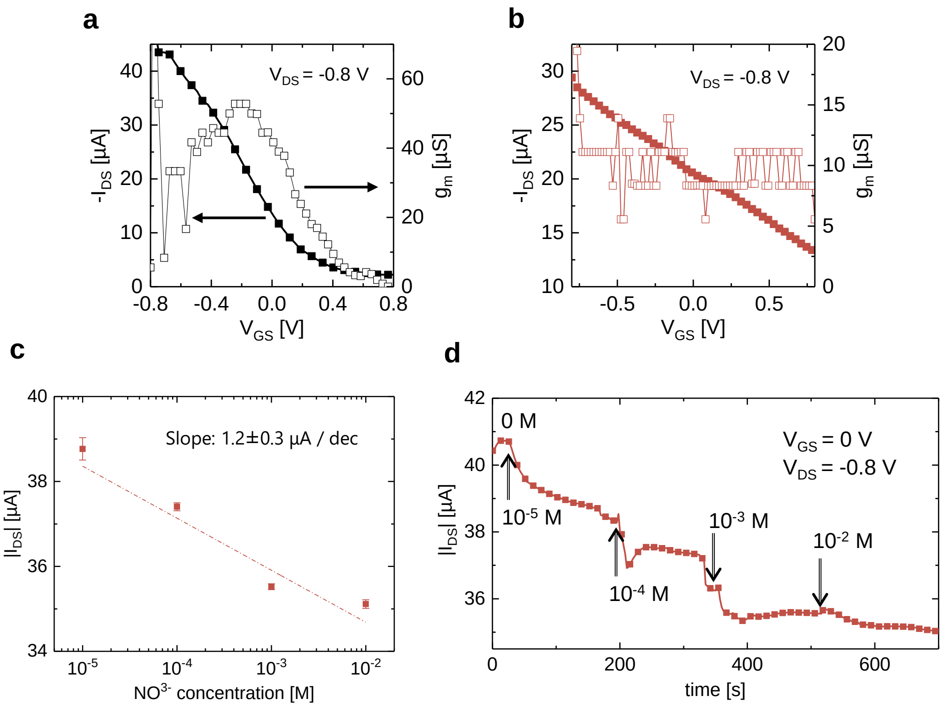

Figure S12. Nitrate ion sensing using OECTs. **(a, b)** Transfer characteristics of OECTs (W/L = 2000/100 µm) measured with Ag/AgCl gate electrode **(a)** and nitrate-ion sensitive electrode **(b)** in diluted saline solution (15 mM). **(c)** Measured steady-state drain current (I_{DS}) as a function of nitrate ion (NO_3^-) concentration in diluted saline solution. **(d)** Real-time traces of I_{DS} in response to an increase in NO_3^- concentration in diluted saline solution at V_{GS} = 0 V and V_{DS} = −0.8 V.

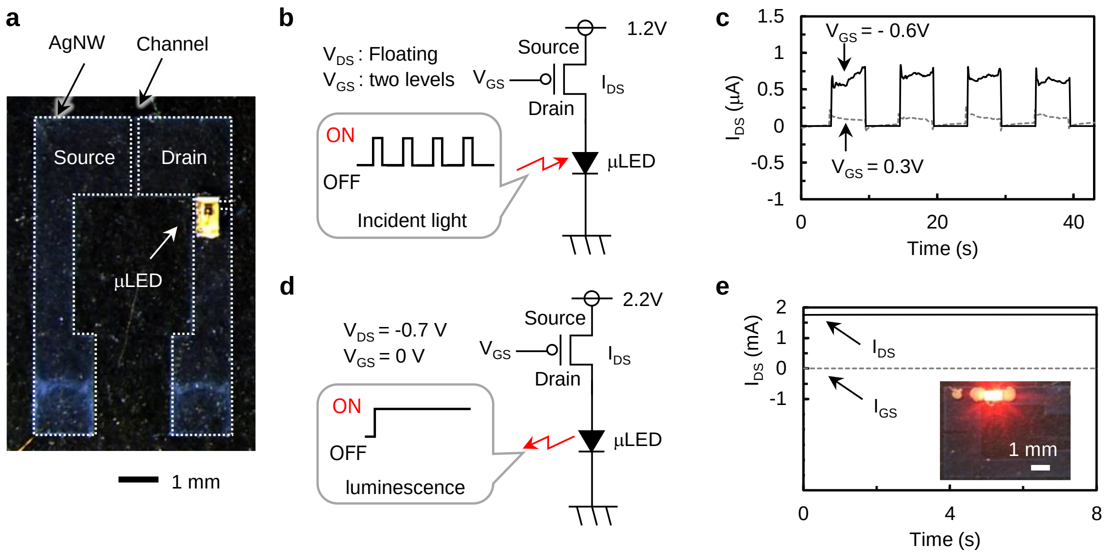

Figure S13. Optical devices using transparent OECT. (**a**) An image of OECTs (W / L = 2000 µm / 100 µm) integrated with a micro-light emitting diode (µLED). The image of the optical micrograph corresponds to the dark field. (**b**) A circuit designed for photo-detection using an OECT functioning as a switch. (**c**) OECT drain current (I_{DS}) under white-colored incident light during ON-state ($V_{GS} = -0.6$ V) and OFF-state ($V_{GS} = 0.3$ V) of the OECT. I_{DS} under ON-state and light illumination were 7-times larger than those of OFF-state. (**d**) A circuit designed for luminescence. (**e**) OECT drain current (I_{DS}) during µLED emission (inset: image of luminescence). Gate leakage (I_{GS}) of 0.6 µA was measured while the µLED was turned on by I_{DS} of 1.75 mA. In the future, the ON/OFF ratio of the OECT should be improved to increase the contrast between light and dark for both optical devices of photo-detector and luminescence. Room temperature cured Ag paste was used when the contact between the AgNW-based wiring and µLED was not stable, and a stable contact method should be considered in the future.

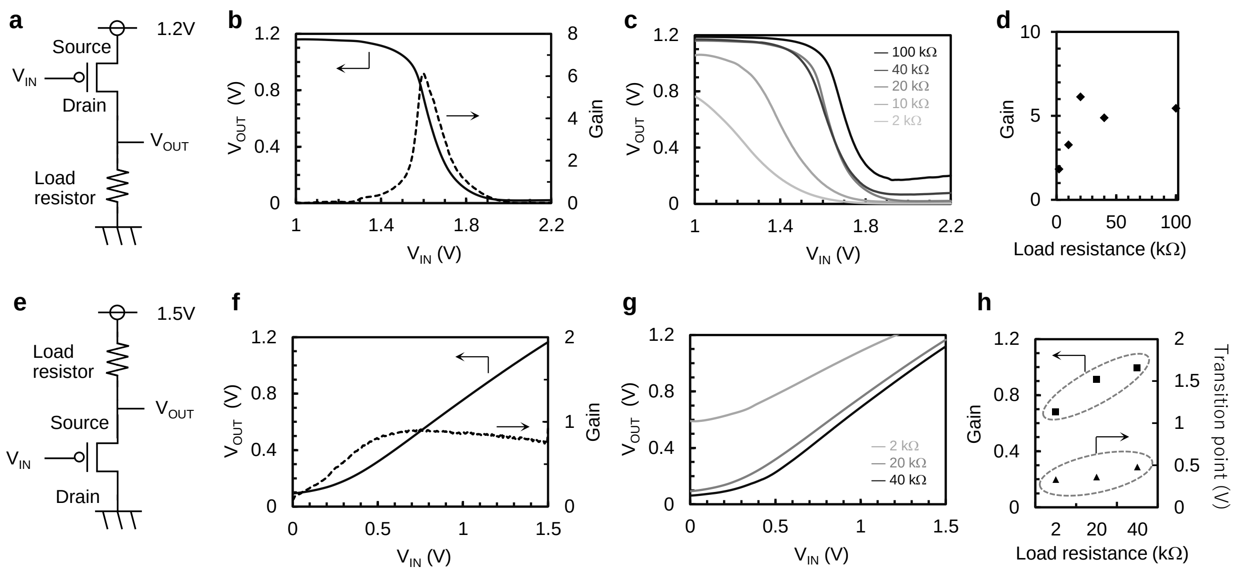

Figure S14. Simple circuits using transparent OECT. **(a)** Circuit diagram of an amplifier using OECT. **(b)** Relationship between input voltage (V_{IN}) and output voltage (V_{OUT}) when a 20 kΩ load resistor in **a** is used. The gain for each V_{IN} is represented on the second vertical axis. **(c)** The input/output characteristics and **(d)** the gain for each load resistance. The channel resistance of the OECT in dry conditions is approximately 20 kΩ, and the highest gain of 6.1 was observed when the load resistance close to that value was used. **(e)** Circuit diagram of a source follower using OECT. **(f)** Relationship between V_{IN} and V_{OUT} when a 20 kΩ load resistor in **e** is used. The gain for each V_{IN} is represented on the second vertical axis. **(g)** The input/output characteristics and **(h)** the gain for each load resistance. The transition point at which the output potential begins to stabilize (−3 dB relative to the maximum gain) increased with the increasing load resistance. The circuit for EEG measurement shown in **Figure 5a** is almost identical to the configuration of a source follower with a load resistance of 20 kΩ and monitored output voltage divider. In addition, an offset voltage of approximately 0.2–0.4 V is generally generated in V_{IN} between the gate for PEDOT:PSS channel that was placed on the forehead (measurement point) and the gel paste used for the mastoid process (ground for the measurement). The offset voltage is likely to provide a stable V_{OUT} in the region above the transition point of the source follower.

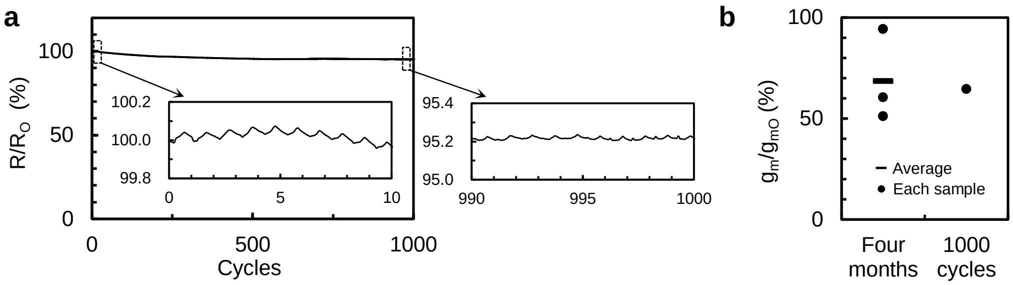

Figure S15. Cyclic bending test of transparent OECT. **(a)** Change in electrical resistance of PEDOT:PSS channel without electrolyte during the 1000-cycle bending test. Inset figures correspond to 0–10 cycles and 990–1000 cycles. The change in resistance was calculated by measured resistance, R divided by resistance before the test, R_O. A mechanical testing machine (EZ test; Shimadzu Co., Kyoto, Japan) was used to bend the transparent OECT (bending radius: approximately 4 mm) and release it back to its initial position during the resistance measurement (34461A; Keysight Technologies, USA). The total time for one cycle is 10 s. **(b)** Change in transconductance (measured valued, g_m divided by one before the storage, g_{mO}) of OECT after 4 months of storage at room temperature and after further cyclic bending tests. Although room temperature curable Ag paste was used at the contact point between the contact pad of the OECT and the wiring for the electrical resistance measuring instrument, destruction occurred in the area below the bending radius tested. Thus, only one of the three samples left was subjected to the bending test. As shown in **Figure 4**, the durability of the OECT was confirmed down to a bending radius of 0.8 mm. In the future, a connection method to stabilize the contact pad of the OECT and the external connection wiring should be investigated.

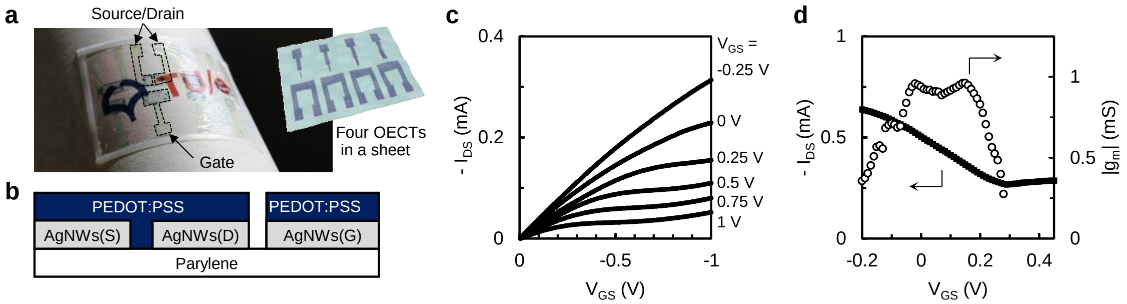

Figure S16. Characteristics of fully transparent ultrathin OECTs as a planar structure. **(a, b)** Conceptual photograph **(a)** and cross-sectional schematics **(b)** on the planar structure. **(c, d)** Output **(c)** and transfer **(d)** characteristics of representative OECTs with channel length and width of 100 and 2000 μm, respectively. The size of the gate for immersion in the electrolyte is 1 × 1 mm. High transconductance (≈1 mS) was observed, which is comparable to the one with Ag/AgCl gate electrode, as shown in **Figure 2(f)**. However, the ON/OFF ratio of OECT is less than 10 for a planar structure and up to 46 for an Ag/AgCl gate electrode. In the future, improvement in trends should be investigated based on the device size of the OECT, the positional relationship between the gate electrode and the channel, etc.
